# HCV NS5A dimer interface residues regulate HCV replication by controlling its self-interaction, hyperphosphorylation, subcellular localization and interaction with cyclophilin A

**DOI:** 10.1371/journal.ppat.1007177

**Published:** 2018-07-23

**Authors:** Saravanabalaji Shanmugam, Alyssa K. Nichols, Dhanaranjani Saravanabalaji, Christoph Welsch, MinKyung Yi

**Affiliations:** 1 Department of Microbiology and Immunology, University of Texas Medical Branch at Galveston, Galveston, Texas, United States of America; 2 Department of Internal Medicine I, Goethe University, Frankfurt/Main, Germany; The University of Chicago, UNITED STATES

## Abstract

The HCV NS5A protein plays multiple roles during viral replication, including viral genome replication and virus particle assembly. The crystal structures of the NS5A N-terminal domain indicated the potential existence of the NS5A dimers formed via at least two or more distinct dimeric interfaces. However, it is unknown whether these different forms of NS5A dimers are involved in its numerous functions. To address this question, we mutated the residues lining the two different NS5A dimer interfaces and determined their effects on NS5A self-interaction, NS5A-cyclophilin A (CypA) interaction, HCV RNA replication and infectious virus production. We found that the mutations targeting either of two dimeric interfaces disrupted the NS5A self-interaction in cells. The NS5A dimer-interrupting mutations also inhibited both viral RNA replication and infectious virus production with some genotypic differences. We also determined that reduced NS5A self-interaction was associated with altered NS5A-CypA interaction, NS5A hyperphosphorylation and NS5A subcellular localization, providing the mechanistic bases for the role of NS5A self-interaction in multiple steps of HCV replication. The NS5A oligomers formed via different interfaces are likely its functional form, since the residues at two different dimeric interfaces played similar roles in different aspects of NS5A functions and, consequently, HCV replication. In conclusion, this study provides novel insight into the functional significance of NS5A self-interaction in different steps of the HCV replication, potentially, in the form of oligomers formed via multiple dimeric interfaces.

## Author summary

HCV NS5A is a multifunctional protein involved in both viral RNA replication and infectious virus production, and is a target of one of the most potent antivirals available to date. However, the mode of action of NS5A inhibitors is still unclear due to the lack of mechanistic detail regarding NS5A functions during HCV life cycles. In this study, we have provided evidence that surface-exposed NS5A residues involved in two different dimeric interactions in crystal structures are indeed involved in NS5A self-interactions in cells. We also showed that these NS5A residues play critical role in HCV RNA replication and infectious virus production by regulating NS5A hyperphosphorylation, its subcellular localization and its interaction with host protein CypA. Overall, our data support the functional significance of “NS5A oligomers” formed via multiple interfaces in HCV replication. We speculate that the NS5A inhibitors exploited the NS5A oligomer-dependent functions during HCV replication, rather than targeting individual NS5A, which consequently resulted in their high potency.

## Introduction

Hepatitis C virus (HCV) is a main causative agent associated with chronic liver diseases including chronic hepatitis, cirrhosis and hepatocellular carcinoma [1, 2]. It is an enveloped, positive-stranded RNA virus belonging to the genus hepacivirus within the flaviviridae family [3]. A single polyprotein translated from the viral genome encodes structural proteins, including core, E1, and E2 at the N-terminal domain followed by the viral assembly accessory proteins p7 [4, 5] and NS2 [6–10]. The C-terminal domain encodes five different nonstructural proteins including NS3, NS4A, NS4B, NS5A and NS5B, which comprise viral replicase complexes [11] and regulate viral assembly [12–17].

NS5A associates with membrane through its N-terminal amphipathic helix (AH) domain [18]. Following the AH domain are three major domains called domain I (DI), DII, and DIII. These domains are separated by two low-complexity sequences (LCS) called LCSI and LCSII. In general NS5A-DI and DII were shown to play roles in HCV RNA replication [19–21], and DIII was associated with virus particle assembly [16]. NS5A is a phosphoprotein expressed as a hypophosphorylated form, which is further phosphorylated to a hyperphosphorylated form [22, 23]. The clusters of highly conserved residues at LCSI served as a target of casein kinase I-α (CKI-α)-mediated hyperphosphorylation, and blocking this inhibited the NS5A localization to the lipid droplets (LD)-associated, low-density membranes and impaired infectious virus production [17]. The casein kinase II-mediated NS5A-DIII phosphorylation was also shown to affect HCV particle assembly by regulating NS5A and core interaction [24, 25].

Highly effective anti-HCV therapies are composed of different combinations of antiviral compounds targeting viral enzymes, such as NS3/4A protease and NS5B polymerase, and NS5A [26]. Since NS5A lacks enzyme activity, NS5A inhibitors were discovered via high throughput screening (HTS) of chemical libraries by using HCV replicon systems [27, 28]. NS5A inhibitors are one of the most potent antivirals to date, inhibiting NS5A function during HCV replication at a picomolar range in cell culture-based systems (reviewed in [29]). NS5A inhibitors impaired HCV RNA replication by preventing the formation of double membrane vesicles (DMV) that constitute HCV RNA replication factories [30–32]. NS5A inhibitors also blocked intracellular HCV particle assembly [33]. Both AH and DI domains of NS5A were indispensable for DMV formation [19]. Therefore, it is no surprise that NS5A inhibitors, which were shown to inhibit DMV formation, target these two domains, as evidenced by the accumulation of drug-resistant mutations in these areas [34]. Interestingly, many NS5A inhibitors are *bivalent* (have two identical pharmacophores), and the bivalent form of compounds such as the iminothiazolidinone-based compound from Bristol-Myers Squibb (BMS-824) are much more potent than the corresponding monovalent forms [27, 35]. These observations fueled the speculation that bivalent NS5A inhibitors target the dimeric form of NS5A. In fact, multiple lines of evidence from different studies support that NS5A functions as a dimer or multimer. These include its dimeric crystal structures [36–38] and the detection of NS5A-NS5A interaction *in vitro* by using purified proteins [20, 39] or *in vivo* in the NS5A ectopic expression system [40, 41] and HCV-replication system [30, 42]. In addition, Lim et al. showed that mutations introduced to four cysteine residues in NS5A (C39, C57, C59 and C80), shown to be involved in Zinc binding and required for HCV RNA replication [43], inhibited purified NS5A self-interaction [20]. However, while these studies clearly demonstrated the correlation between NS5A dimerization and HCV RNA replication, the exact roles of NS5A dimerization in HCV life cycle is still unclear.

In this study, we performed a structure-function analysis of highly conserved, surface-exposed NS5A-DI residues located at two different dimer interfaces, predicted from NS5A-DI crystal structures, on NS5A protein interactions and HCV replication. Our data indicate that these dimer interface residues are involved in NS5A self-interaction in Huh-7 cells and that NS5A self-interactions through these residues are critical for different steps of HCV replication by regulating NS5A hyperphosphorylation, subcellular localization and interaction with host protein CypA.

## Results

### Mutations introduced to two different interfaces of NS5A dimeric forms inhibited NS5A self-interaction in Huh-7 cells

**Fig 1:**
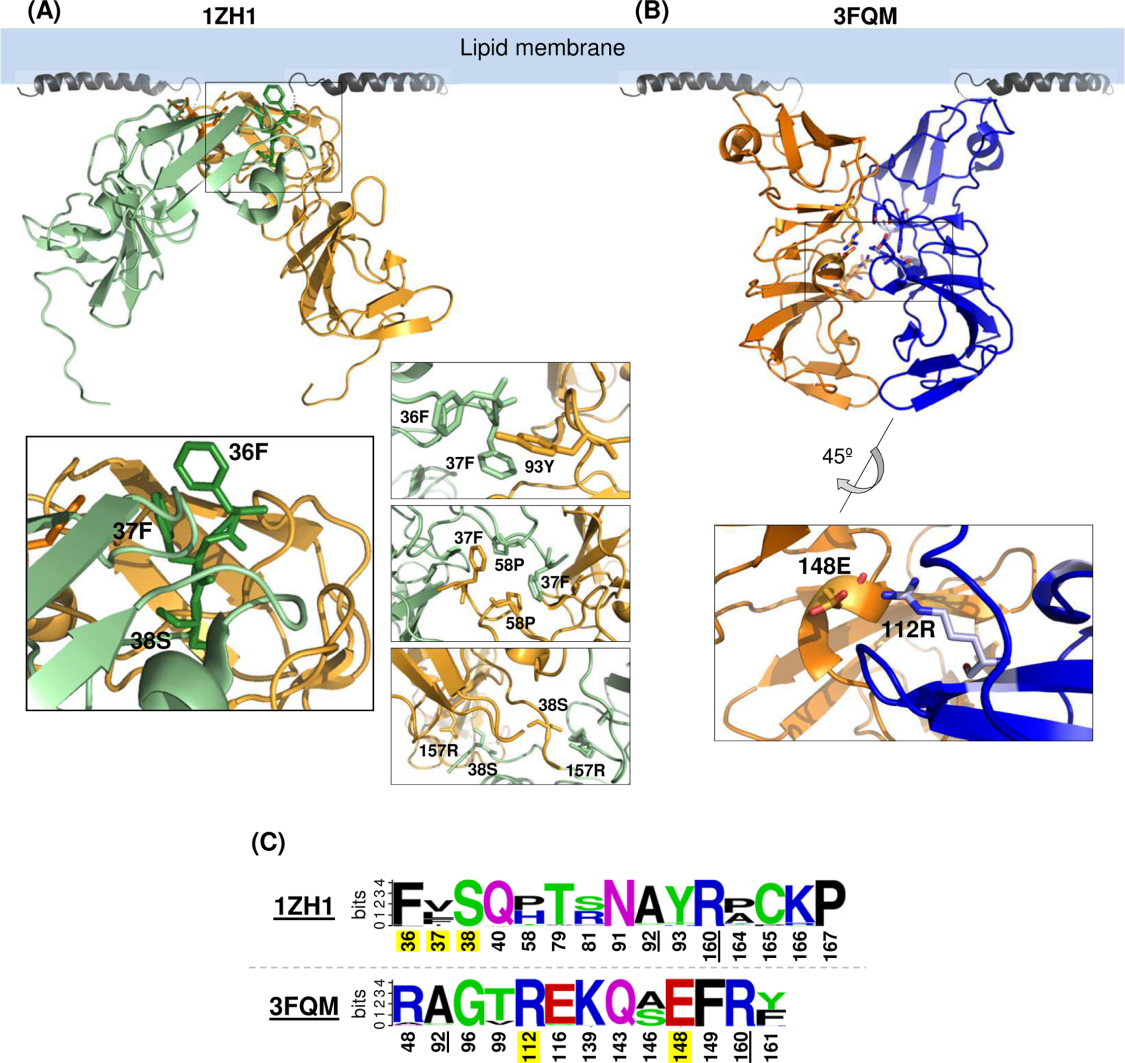
Two different crystal structures of genotype 1b NS5A-DI. Two NS5A-DI structures including (A) PDB-1ZH1 and (B) PDB-3FQM depicted below the NMR structure of the N-terminal amphipathic helix (PDB-1R7G) to show their relative orientation to the membrane. At the bottom are the enlarged images showing the potential interaction of dimer interface residues analyzed in this study. (C) The sequence logo depiction of amino acid conservation in NS5A residues lining the 1ZH1 and 3FQM dimer among 672 HCV sequences derived from all genotypes deposited in the Los Alamos HCV database. Common residues are underlined. The residues analyzed in this study are highlighted in yellow. The overall height of the stack represents the sequence conservation at that position and the height of individual amino acid residues within the stack show their relative frequency. The polar amino acids (G, S, T, Y, C, Q, N) are shown in green, basic (K, R, H) in blue, acidic (D, E) in red and hydrophobic (A, V, L, I, P, W, F, M) in black.

The genotype 1b (gt1b) NS5A-DI crystal structures depicted in Fig 1, designated as 1ZH1 and 3FQM, suggested that the NS5A self-interaction occurs via at least two different interfaces [36, 37]. To determine the relevance of these two dimeric forms of NS5A in its self-interaction in cells, we performed alanine-scanning mutagenesis of selected dimer-interface residues in full- length NS5A derived from gt1a (H77 strain) and gt2a (JFH1 strain) and determined the impact of these mutations on NS5A self-interaction. NS5A-DI residues 36, 37 and 38 were chosen since they provide a continuous surface patch involved in 1ZH1-specific dimer interaction (Fig 1A, see also S1A Fig showing the 1ZH1 residue interaction networks for detail). Residues 112 and 148 were selected since they could form a salt bridge at the 3FQM-specific dimer interface (Fig 1B, see also S1B Fig showing the 3FQM residue interaction networks for detail). As shown in Fig 1C, residues 38 serine (38S), 112 arginine (112R) and 148 aspartic acid (148E) are completely conserved among 672 HCV sequences derived from all genotypes deposited in the Los Alamos HCV database. Residue 36 encodes major variant phenylalanine (F) or minor variant leucine (L). Residue 37 encodes hydrophobic residues, including valine (V), leucine (L), phenylalanine (F) or isoleucine (I).

**Fig 2:**
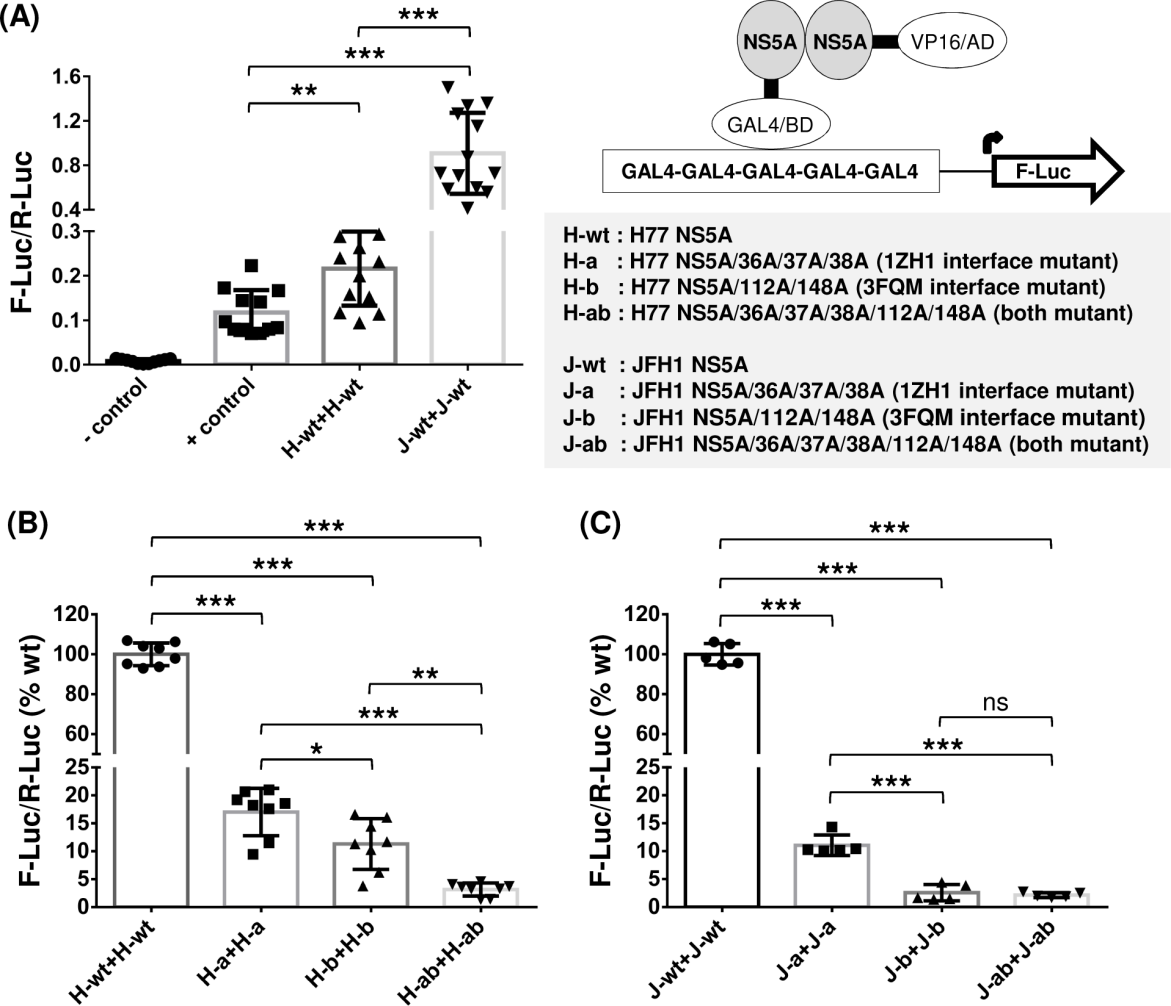
Two different NS5A dimer-interface mutations impaired both gt1a H77 and gt2a JFH1 NS5A self-interactions. (A) An NS5A self-interaction was determined by performing a checkmate mammalian two-hybrid assay in Huh-7 cells. Interaction between GAL4/BD (GAL4 DNA binding domain)-fused protein and VP16/AD (VP16 activation domain)-fused protein resulted in increased F-Luc (Firefly luciferase) activity under the control of five GAL4-binding sites, which was then normalized with R-Luc (Renilla luciferase encoded in GAL4/BD vector) activity to adjust the transfection efficiency. The F-Luc/R-Luc value from a vector-only transfected sample is used as a negative (-) control and that from the well-known interaction partner ID and MyoD was used as a positive (+) control of this assay. Both gt1a H77 and gt2a JFH1 NS5A showed self-interactions that are stronger than the (+) control. (B and C) Mutations introduced to either 1ZH1 or 3FQM or both dimeric interfaces impaired the self-interaction of gt1a H77 NS5A (B) and gt2a JFH1 NS5A (C). The percentages of F-Luc/R-Luc ratios, relative to wt NS5A self-interaction, were presented. The results are from three to five independent experiments. Asterisks indicate statistically significant differences between paired values: ***, *P* <0.0005; **, *P* <0.005; and *, *P* <0.05. The differences with a *P* ≥0.05 were considered not significant (ns).

We determined the NS5A self-interaction by using a mammalian two-hybrid system as previously described [40, 41] (Fig 2). In brief, NS5A was fused to the GAL4 DNA-binding domain (GAL4/BD) in pBIND vector and the herpes simplex virus VP16 activation domain (VP16/AD) in pACT vector. Then, these two vectors plus a third vector, pGL4.3[*luc2P*/*Gal4*UAS/Hygro], encoding five GAL4 binding sites upstream of the firefly luciferase (F-Luc) gene, were transfected to Huh-7 cells. Two days following the transfection of these plasmids, cells were lysed to detect NS5A self-interaction efficiency by measuring F-Luc activity normalized by that of Renilla reniformis luciferase (R-Luc) expressed from a pBIND vector to adjust transfection efficiency. Under these experimental conditions, stronger protein-to-protein interaction resulted in higher F-Luc/R-Luc ratio. These ratios derived from the basal vectors (pACT and pBind) and the known interacting-pair [pACT-MyoD (myogenic regulatory protein) and pBind-ID (negative regulator of myogenic differentiation)] were used as a protein-protein interaction negative (-) control and positive (+) control, respectively (Fig 2A). Robust interaction was detected between a wild-type (wt) NS5A pair derived from a gt1a HCV H77 (designated as H-wt), evidenced by the higher F-Luc/R-Luc value from H-wt pair than that from the (+) control. Interestingly, NS5A from a gt2a HCV JFH1 (designated as J-wt) showed an even stronger self-interaction than did H-wt (Fig 2A). Next, we determined the impact of alanine mutations in the residue lining the 1ZH1 and 3FQM NS5A dimer interfaces, including 36A/37A/38A (mutation group a) and 112A/148A (mutation group b), respectively. These H77 NS5A mutants, designated as H-a or H-b, showed a significantly reduced NS5A-NS5A interaction compared to that of H-wt (Fig 2B). The mutants having both mutations, designated as H-ab, showed a stronger reduction in NS5A self-interaction (Fig 2B). Similarly, JFH1 NS5A-NS5A interaction was also significantly reduced when these mutations were introduced separately or combined (Fig 2C). In summary, these data suggest that NS5A from gt1a H77 and gt2a JFH1 form dimers/oligomers via at least two different dimeric interfaces in Huh-7 cells.

### Altered H77 NS5A self-interaction impaired gt1a H77D virus RNA replication

**Fig 3:**
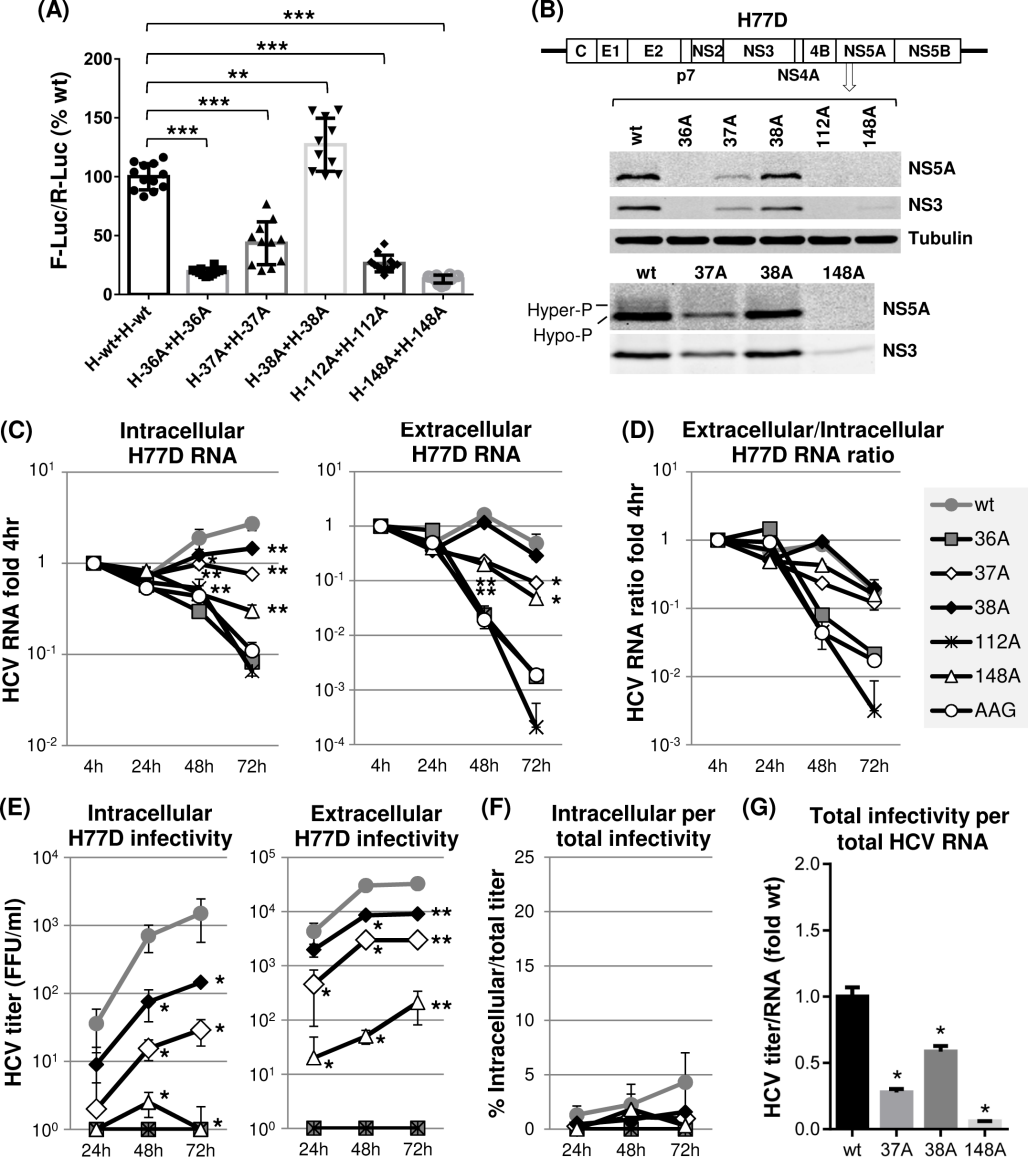
The effects of individual dimer interface mutations on H77 NS5A self-interaction and gt1a H77D replication. (A) The mammalian two-hybrid assay was performed to detect the impact of individual mutations on H77 NS5A self-interaction. Results are from three-to-four independent experiments. The percentages of F-Luc/R-Luc ratios relative to wt NS5A self-interaction were presented. (B) Diagram of gt1a H77D at the top with different NS5A mutations. The middle panel shows the western blot detection of NS5A, NS3 and tubulin in Huh-7 cell lysates prepared at day 3 post-electroporation of H77D RNA to these cells. The bottom panel presents the western blot results of the same lysates with increased amounts to detect potential hyperphosphorylation of H77 NS5A. (C) The left and right panels show the results of qRT-PCR performed to detect HCV RNA present in cell lysates (intracellular) or cell culture supernatants (extracellular), respectively, collected at different time point post-electroporation of H77D RNA with or without indicated mutations. The RNA percentage values reactive to the 4 h level are shown. AAG indicates replication-defective H77 NS5B polymerase mutant. (D) HCV RNA ratios between extracellular and intracellular RNA calculated relative to 4 h values. (E) The left and right panels showed the results of virus titration by using cell lysates (intracellular) and cell culture supernatants (extracellular), respectively, at different time point post-electroporation of H77D RNA with or without indicated mutations. (F) The percentage of intracellular virus titers per total (intracellular plus extracellular) virus titer. (G) The ratio of total titer per total HCV RNA level relative to that wt H77D.

To narrow down the critical residues involved in H77 NS5A self-interaction, we introduced individual alanine mutations to residues 36, 37, 38, 112 or 148 in H77 NS5A two-hybrid vectors and designated them as H-36A, H-37A, H-38A, H-112A or H-148A, respectively. Then the effects of these individual mutations on NS5A self-interaction were determined by measuring the dual luciferase (F-Luc and R-Luc) activities from Huh-7 cells transfected with these plasmids as described above. As shown in Fig 3A, all mutations, with the exception of 38A, significantly reduced NS5A self-interaction down to the levels between ~10 to 50% of wt H77 NS5A-NS5A interaction efficiency on average. Interestingly, the 38A mutation significantly enhanced NS5A self-interaction (~30% above wt level on average). We speculate that 38A mutation altered the non-covalent interactions of a residue 157 arginine (157R), which was a wt 38S interactor, inadvertently stabilizing the NS5A inter-molecular interaction (see S1 Fig). Next, we determined the effect of mutations modulating NS5A self-interaction on infectious gt1a H77D replication [44], following electroporation of wt and mutant H77D RNAs to Huh-7 cells, then, determining the expression of viral proteins and RNAs at different time points post electroporation by western blot and quantitative RT-PCR analyses (Fig 3B and 3C). Results indicated that altered H77 NS5A self-interaction inhibited viral replication (Fig 3B and 3C). In detail, the 37A and 148A mutations caused moderate-to-substantial reductions in viral protein expression and RNA replication (Fig 3B and 3C). The 36A and 112A mutations completely blocked viral replication as evidenced by undetectable viral protein expression and the lack of viral RNA increase over the replication-defective H77 mutant AAG [45] (Fig 3B and 3C). The 38A mutation, which moderately enhanced NS5A self-interaction, also reduced HCV RNA replication (Fig 3C, left panel). These data suggest that optimal mode of H77 NS5A self-interaction is critical for efficient gt1a virus RNA replication. Alternatively, 38A mutation could have disrupted other functions of H77 NS5A unrelated to NS5A self-interaction, or altered overall conformation of NS5A, resulting in H77D replication defect. To better understand the exact role of H77 NS5A residue 38 in HCV RNA replication, we have tried to obtain the revertant of H77D/38A mutant. However, our multiple attempts to obtain H77D/38A revertant were unsuccessful.

### H77 NS5A self-interaction was critical for efficient gt1a H77D particle assembly but not for the density or specific infectivity of secreted virions

Next, to test the effect of H77 NS5A self-interaction-altering mutations on infectious virus production, we determined the intracellular and extracellular infectivity titers following electroporation of H77D RNA having these individual mutations to Huh-7 cells. As expected, replication-defective H77D/36A or H77D/112A mutants showed no evidence of virus production (Fig 3E). Other mutants, including H77D/37A, H77D/38A and H77D/148A showed severely impaired virus production ranging between ~10- to ~1,000-fold reductions in intracellular infectivity and ~3- to ~100-fold reductions in extracellular infectivity compared to those of the wt at the 72-h time point (Fig 3E). None of the virus-producing H77 NS5A mutants impaired the secretion of virus particles, since the percentage of intracellular infectivity per total (intracellular plus extracellular) infectivity was similar between the wt H77D and these mutants (Fig 3F). Supporting this, the ratio of extracellular and intracellular HCV RNA was similar between the wt H77D and its virus-producing NS5A mutants (Fig 3D). On the other hand, virus particle-assembly efficiency was significantly reduced in these H77 NS5A mutants, since the relative levels of total infectivity per total HCV RNA in these mutants were significantly lower than that from wt H77D (Fig 3G).

**Fig 4:**
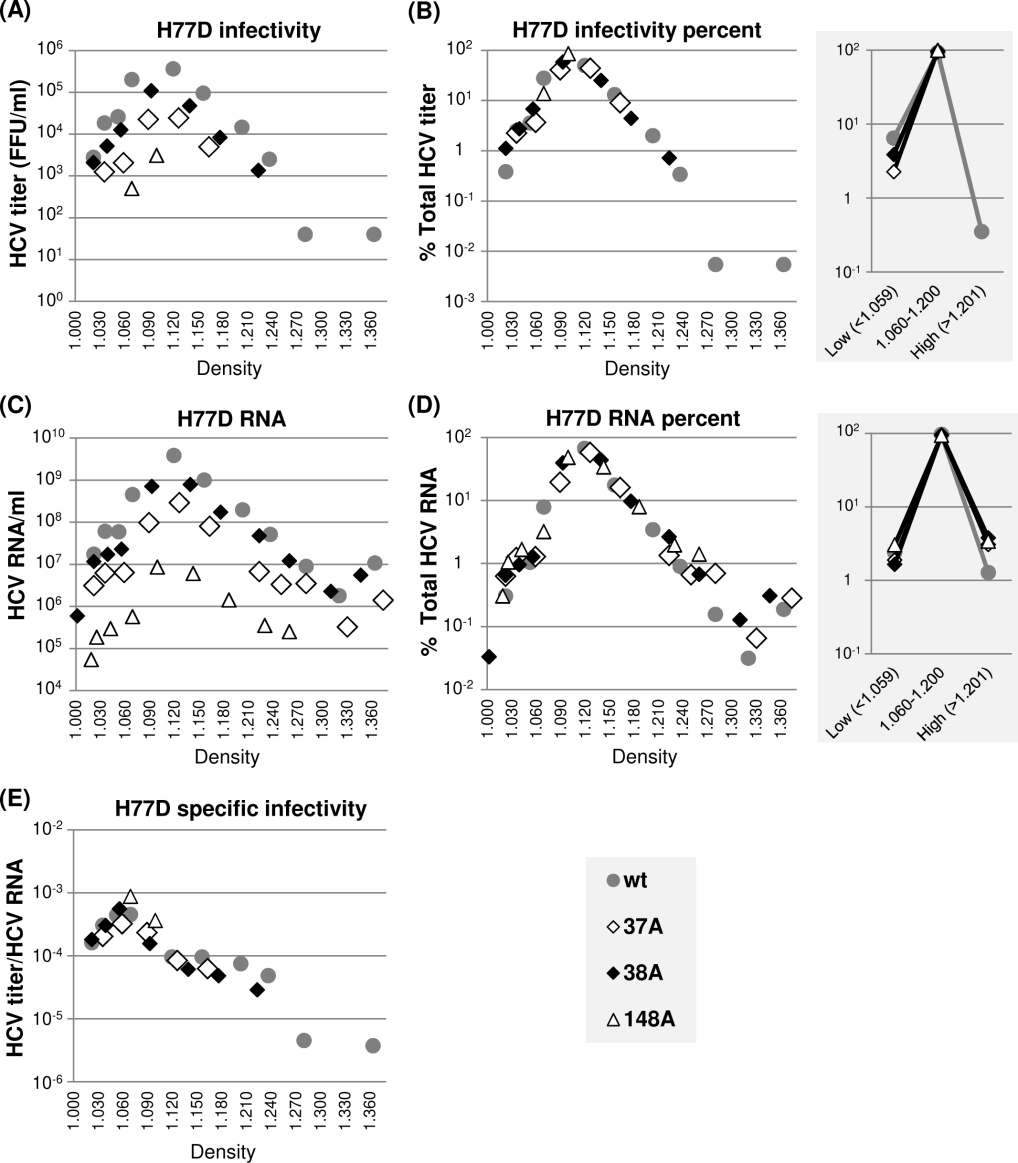
NS5A dimer interface mutations did not affect H77D virus particle density or specific infectivity. (A) Distribution of infectious virus present in different density fractions following equilibrium ultracentrifugation of cell culture supernatants collected between 48 h and 72 h post-electroporation of H77D and indicated mutants to Huh-7 cells. (B) The percentages of virus titers present at different density fractions relative to total titers from individual viruses are shown at left and the percentages of virus titers present in three different density ranges are presented at right. (C) Distribution of HCV RNA at different density fractions as determined by qRT-PCR. (D) The percentages of HCV RNA levels present at different density fractions relative to total HCV RNA from individual H77D derivatives are shown at left and the HCV RNA percentages present in three different density ranges are shown at right. (E) The distribution of virus-specific infectivity, calculated as the ratio of virus titers and HCV RNA distribution at different density fractions.

Next, we performed a density gradient centrifugation of extracellular virus present in the cell culture supernatant to determine whether H77 NS5A mutations, including 37A, 38A and 148A, impaired the H77D infectivity by modulating virus particle density or specific infectivity. The results shown in Fig 4 indicate that the density distributions of wt H77D and three different NS5A mutant viruses are similar, since the relative percentages of HCV RNA and infectivity present in different density fractions are almost identical between the wt and mutants (Fig 4B and 4D). We also did not detect any significant alteration in the specific infectivity of virus particles between the wt and mutants calculated as a ratio of infectivity per HCV RNA at different density fractions (Fig 4E). Interestingly, the majority of infectious particles (>95%) from both wt and mutants banded at a density between 1.060 and 1.200 g/cm^3^, centering around at 1.100 g/cm^3^ (Fig 4B), whereas the peak of specific infectivity was detected at a density of ~1.060 g/cm^3^ (Fig 4E). These data indicate that a small fraction of low-density gt1a virus particles is more infectious than the majority of virus particles, consistent with previous reports [46, 47].

In summary, these results suggest that H77 NS5A self-interaction is critical for infectious particle-assembly efficiency, but has little impact on specific infectivity and density of infectious virus particles as well as their egress efficiency.

### Genotypic differences in the role of individual dimer interface mutations on NS5A self-interaction and HCV RNA replication

**Fig 5:**
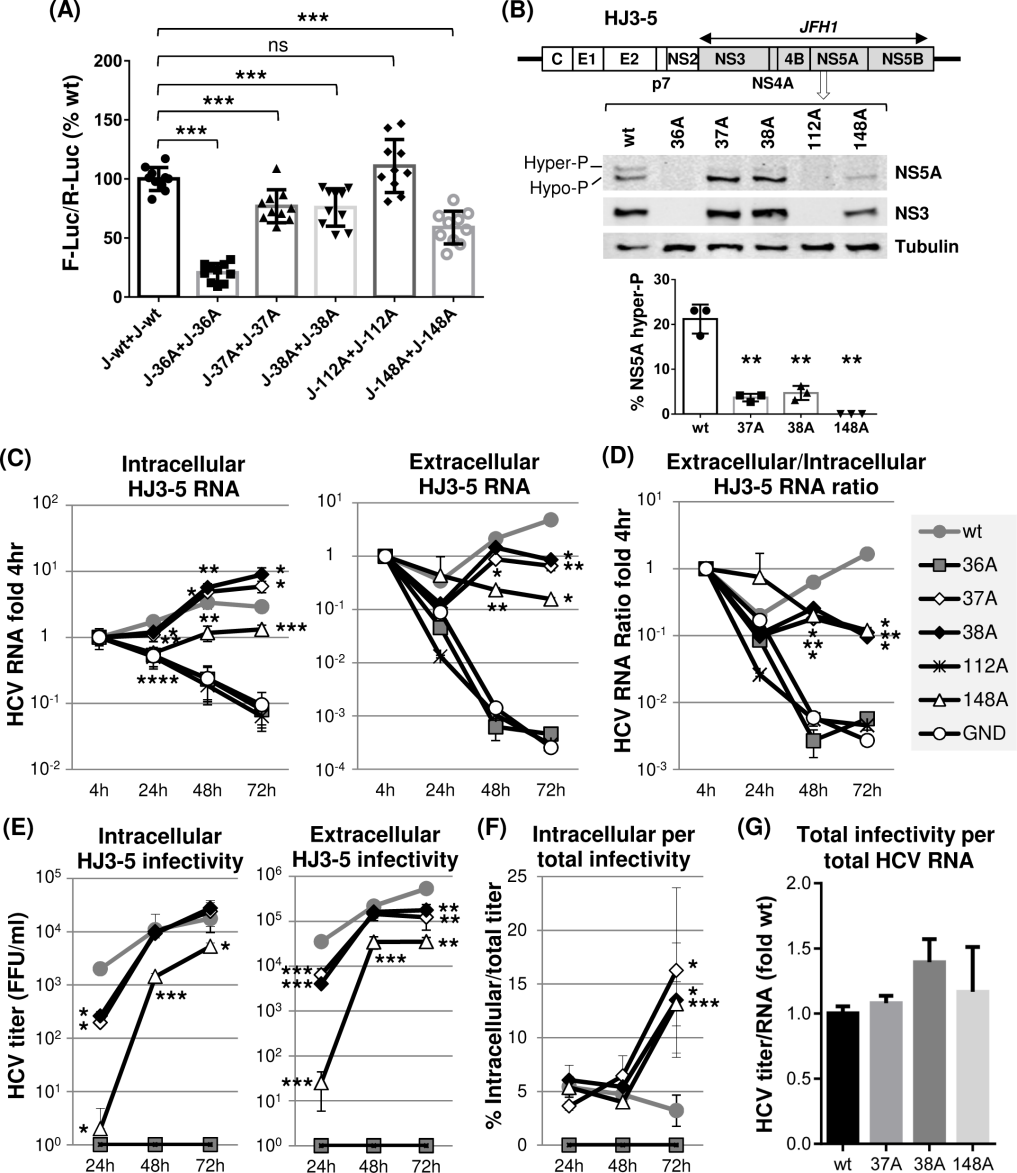
The effects of individual dimer interface mutations on JFH1 NS5A self-interaction and HJ3-5 RNA replication. (A) The checkmate mammalian two-hybrid assay was performed to detect the impact of individual mutations on JFH1 NS5A self-interaction. Results are from three-to-four independent experiments. The percentages of F-Luc/R-Luc ratios relative to wt NS5A self-interaction were presented. (B) Diagram of gt1a/gt2a chimera HJ3-5 at the top with different NS5A mutations. Shaded protein coding sequences were derived from gt2a JFH1. The middle panel shows the western blot detection of NS5A, NS3 and tubulin in Huh-7 cell lysates prepared at day 3 post-electroporation of HJ3-5 RNA to these cells. The bottom panel shows the percentage of hyperphosphorylated NS5A detected from three independent experiments. (C) Left and right panels present the results of qRT-PCR performed to detect HCV RNA in cell lysates (intracellular) or cell culture supernatants (extracellular), respectively, collected at different time points post-electroporation of HJ3-5 RNA with or without indicated mutations. The RNA percentage values relative to 4 h level were shown. GND indicates replication-defective JFH1 NS5B polymerase mutant. (D) HCV RNA ratios between extracellular and intracellular RNA calculated relative to the 4 h values. (E) Left and right panels show the virus titration results by using cell lysates (intracellular) and cell culture supernatants (extracellular), respectively, at different time points post-electroporation of HJ3-5 RNA with or without indicated mutations. (F) Percentage of intracellular virus titers per total (intracellular plus extracellular) virus titer. (G) The ratio of total titer per total HCV RNA level. The ratios relative to that of wt HJ3-5 are presented.

We introduced individual alanine mutations to residues 36, 37, 38, 112 or 148 in JFH1 NS5A two-hybrid vectors and designated them as J-36A, J-37A, J-38A, J-112A and J-148A, respectively. We transfected these two-hybrid plasmid sets for each mutant to Huh-7 cells to determine the impact of these individual mutations on JFH1 NS5A self-interaction. It is interesting that most of the mutations introduced individually to JFH1 NS5A showed relatively moderate defects in NS5A self-interaction, retaining between ~60 to 75% of wt JFH1 NS5A self-interaction on average, except for an 36A mutation, which caused severe defects in NS5A self-interaction (~20% of wt interaction). Unlike the 38A mutation in H77 NS5A, which increased NS5A self-interaction, the same mutation in JFH1 NS5A reduced NS5A self-interaction. In the structural perspective, the key inter-domain interactor of wt residue 38S in H77 NS5A is 157R, which provides both van der Waals and hydrogen-bond interactions to 38S (S1A Fig). However, in JFH1, the corresponding 38S interactor is 157 glutamine (157Q), which shows substantial physico-chemical differences from 157R. We speculate that different properties of residue 157 in H77 and JFH1 contributed a genotype-specific difference in the intermolecular interaction phenotypes of 38A mutation. Also contrary to 112A mutation in H77 NS5A, which caused severe defects in NS5A self-interaction, the equivalent mutation in JFH1 NS5A did not impair NS5A self-interaction in cells (compare Figs 3A and 5A). The wt residue 112R in H77 NS5A is involved in a network of electrostatic interactions (“salt bridge” pattern) with the residues 48 arginine (48R) and 148 glutamic acid (148E) in the partnering NS5A resulting in intra/inter-molecular 48R-148E-112R interaction network (S1B Fig). We speculate that the disruption of this electrostatic network mediated by the 112A mutation was substantial enough to impair the intermolecular H77 NS5A self-interaction. However, since the residue 48 alanine (48A) in JFH1 NS5A does not support a similar kind of interaction network, it is reasonable to assume that a different kind of, genotype-specific interaction network surrounding the residue 112 may have negated the impact of 112A mutation on JFH1 NS5A self-interaction. Alternatively, R112 residue in JFH1 NS5A may not contribute to NS5A self-interaction unlike the same residue in H77 NS5A (see discussion).

To determine the effect of these individual JFH1 NS5A mutations on viral RNA replication, we introduced them to H77-JFH1 chimeric HCV (HJ3-5) encoding JFH1 NS3-NS5B proteins [6, 48] (Fig 5B) and then analyzed viral protein expression and HCV RNA levels following electroporation of these RNAs to Huh-7 cells. The results of these experiments could be summarized as follows. First, viral RNA replication was undetectable for HJ3-5/36A mutant (Fig 5B and 5C). Thus, a 36A mutation in NS5A, which impaired both H77 and JFH1 NS5A self-interaction, blocked both H77 and JFH1 RNA replication (Figs 3 and 5). We also detected reduced replication of an HJ3-5/148A mutant, similar to the case of an H77D/148A mutant. The relatively moderate impact of 148A mutation on HJ3-5 replication, compared to its more severe effect on H77D replication, correlates with its weaker impact on self-interaction of JFH1 NS5A compared to that of H77 NS5A (compare Figs 3 and 5). Overall, these data indicate that JFH1 NS5A self-interaction is also critical for JFH1 replicase-mediated viral RNA replication. Second, both 37A and 38A mutants moderately increased the relative levels of intracellular HCV RNA compared to that of wt HJ3-5 at 72 h post electroporation (Fig 5C, left panel). These results may indicate that these two mutations in JFH1 NS5A potentially enhanced HCV RNA replication. However, additional data indicate that this may not be the case. For example, we detected much lower levels of extracellular HCV RNAs from these mutants compared to those from the wt HJ3-5 (Fig 5C, right panel). In addition, relative extracellular/intracellular HCV RNA ratios from these mutants were over 10-fold lower than that from the wt at the 72-h time point (Fig 5D). Based on these data, we believe that decreased viral RNA secretion, rather than enhanced viral replication, has caused the relatively high levels of intracellular 37A or 38A mutant RNA accumulation. Third, an 112A mutation in JFH1 NS5A completely blocked HCV replication despite having no effect on its self-interaction. At a first glance, these data seem to contradict to the potential role of NS5A self-interaction in HCV replication However, previous study determined that HCV replication defect caused by 112A mutation could be attributed to the inhibition of NS5A-RNA interaction and dysregulation of NS5A’s role in HCV translation [49]. Fourth, the 37A, 38A and 148A mutations impaired the JFH1 NS5A hyperphosphorylation (Fig 5B). This phenotype was also observed from the corresponding H77 NS5A mutants, although the hyperphosphorylation efficiency of H77 NS5A was quite low compared to that of JFH1 NS5A (compare Figs 3B and 5B, see also [50, 51]).

In summary, these data indicate that JFH1 NS5A self-interaction also plays an important role in JFH1 replicase-mediated RNA replication, despite some genotype-specific differences.

### Mutations that reduced JFH1 NS5A self-interaction inhibited virus egress but showed little impact on infectious particle assembly

As expected from reduced viral RNA replication, the HJ3-5/148A mutant showed significantly reduced intracellular and extracellular infectivity during the entire time course of experiments (Fig 5E). In the case of 37A or 38A mutants, while they also showed significantly lower intracellular and extracellular titers at the 24-h time point, by 48 h, their titers rapidly caught up with those from the wt HJ3-5. However, by the 72-h time point, extracellular infectivity from these two mutants was significantly lower than that from the wt, while intracellular infectivity remained similar to that of the wt (Fig 5E). The extracellular/intracellular RNA ratios of the 37A, 38A and 148A mutants were also significantly lower than that of wt HJ3-5 at the 48- and 72-h time points (Fig 5D). In addition, the percentages of intracellular infectivity per total infectivity of all three mutants were significantly higher than that of the wt at 72 h (Fig 5F). These results suggested a decreased egress of these mutant viruses compared to the wt virus. Interestingly, none of these mutants affected virus particle-assembly efficiency, since the relative ratios of total HCV infectivity per total HCV RNA were similar between wt and these mutants (Fig 5G). Overall, it is remarkable that same mutations introduced to highly conserved residues 37, 38 and 148 in NS5A showed genotype-specific effects on virus production in that H77 NS5A mutants impaired virus assembly, not viral egress, but JFH1 NS5A mutants impaired viral egress, not virus assembly (Figs 3 and 5).

**Fig 6:**
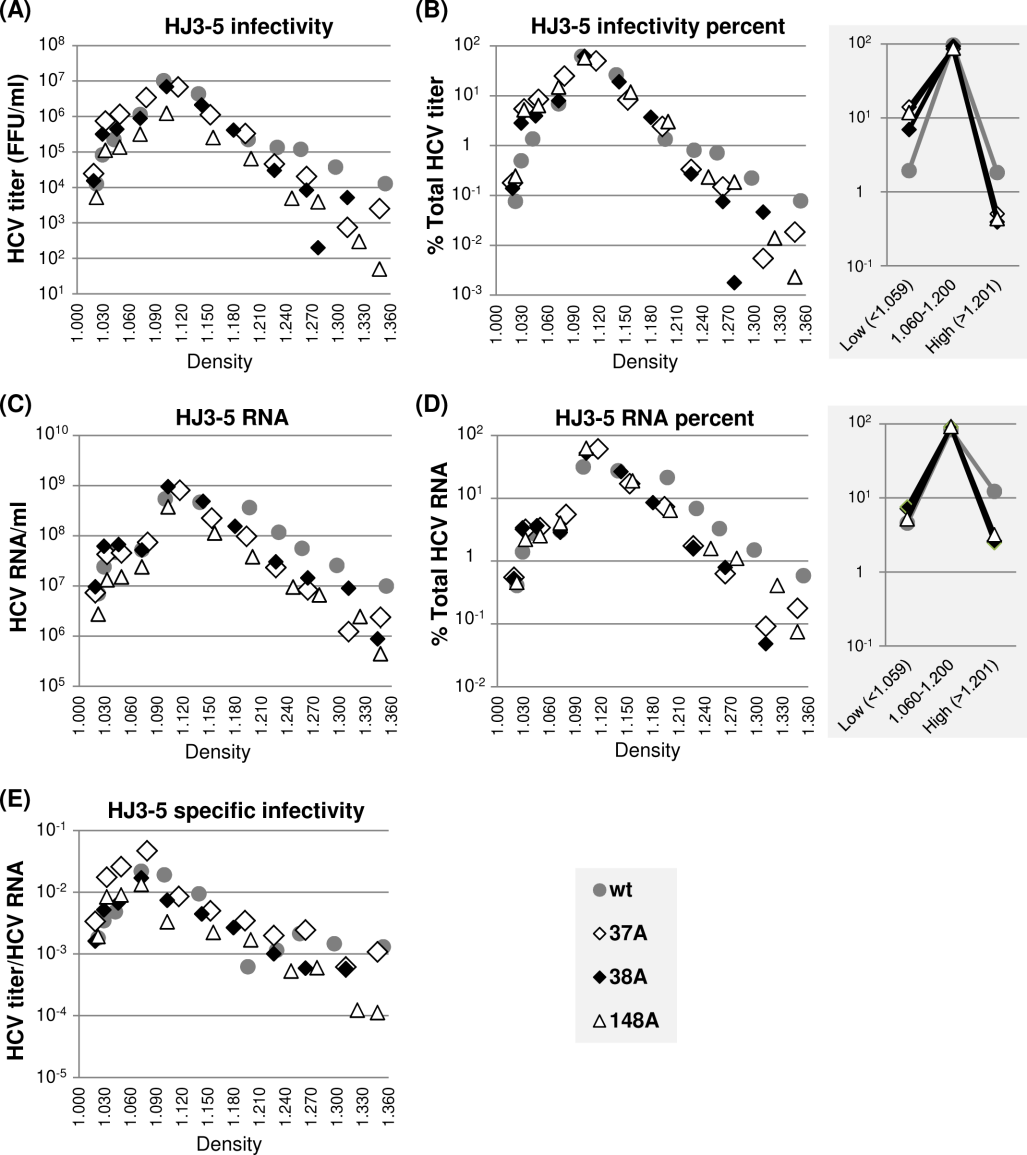
The effects of NS5A dimer interface mutations on HJ3-5 virus particle density distribution. **(**A) Distribution of infectious virus present in different density fractions following equilibrium ultracentrifugation of cell culture supernatants collected between 48 h and 72 h post-electroporation of HJ3-5 and indicated mutants to Huh-7 cells. (B) The percentages of virus titers present at different density fractions relative to total titers from individual viruses are shown at left and the percentages of virus titers present in three different density ranges at right. (C) Distribution of HCV RNA at different density fractions as determined by qRT-PCR. (D) The percentages of HCV RNA levels present at different density fractions relative to total HCV RNA from individual HJ3-5 derivatives are shown at left and the HCV RNA percentages present in three different density ranges at right. (E) The distribution of virus specific infectivity, calculated as the ratio of virus titer and HCV RNA, distribution at different density fractions.

Next, we determined the density and specific infectivity of extracellular HJ3-5 wt as well as those of 37A, 38A and 148A mutant viruses by performing density gradient centrifugation. In general, comparable density distribution patterns were detected between wt HJ3-5 and mutant viruses, as judged from the relative density distributions of viral RNA and infectivity (Fig 6). However, the percentage of wt HJ3-5 in high-density fractions (>1.201 g/cm^3^) was higher than those of mutants. In fact, ~12% of viral RNA from wt HJ3-5 was detected in these high-density fractions compared to ~3% from each of the JFH1 NS5A mutants (Fig 6D, right panel). However, infectivity of wt HJ3-5 in these high-density fractions was low accounting for less than 2% of total infectivity (Fig 6B, right panel). These results suggest that a significant portion of poorly infectious, high-density immature particles might have been secreted from wt HJ3-5-replicating cells, probably due to highly efficient virus egress (Fig 5F, see discussion). On the other hand, relative titers of mutant viruses at low-density fractions (<1.059 g/cm^3^) were 6- to 7-fold higher than those of the wt HJ3-5 (Fig 6B, right panels). Due to this, the specific infectivity of mutant viruses at low-density fractions was relatively higher than that of the wt HJ3-5 (Fig 6E). These results suggest that too efficient virus egress might have negative impact on infectious virus maturation (see discussion).

### Defective NS5A self-interaction impaired NS5A-CypA interaction

**Fig 7:**
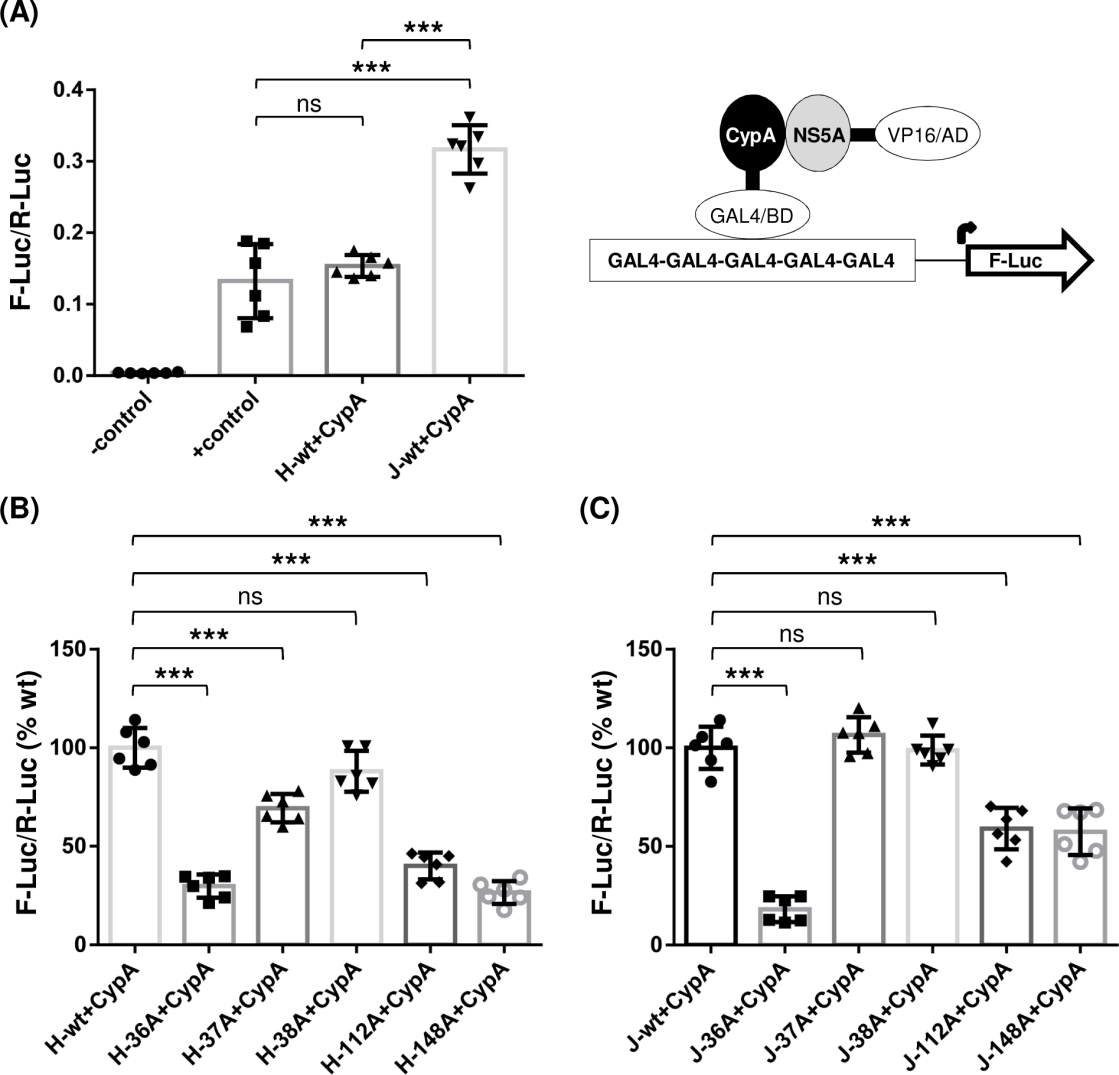
The effects of NS5A dimer interface mutations on NS5A and CypA interaction. **(**A) Interaction between CypA and H77 NS5A or JFH1 NS5A detected by performing a checkmate mammalian two-hybrid assay. Results are from three independent experiments. The effects of individual dimer interface mutations on interaction between CypA and H77 NS5A (B) or JFH1 NS5A (C).

The interaction between NS5A and CypA is critical for HCV RNA replication [52]. Since most of the NS5A self-interaction mutants, especially those derived from gt1a H77D, significantly impaired HCV RNA replication, we asked whether reduced NS5A self-interactions in these mutants might have impaired NS5A-CypA interactions, resulting in decreased HCV RNA replication. To measure the interaction between NS5A and CypA quantitatively, we used a checkmate assay as this method successfully measured the interaction between NS5A and CypA in the previous study [41]. As shown in Fig 7A, the level of interaction between H77 NS5A and CypA was comparable to that of the positive (+) control. Interestingly, interaction between JFH1 NS5A and CypA was stronger than that between H77 NS5A and CypA (Fig 7A). Next, we determined the interaction between CypA and H77 NS5A dimer-interface mutants. The results showed that the mutations that significantly reduced NS5A-NS5A interaction, including H-36A, H-37A, H-112A and H-148A (Fig 3A), also significantly impaired the NS5A-CypA interaction (Fig 7B). These results indicate that effective H77 NS5A self-interaction is critical for H77 NS5A and CypA interaction. These data also suggest that reduced NS5A-CypA interaction in H77 NS5A mutants was responsible for defective viral RNA replication (Fig 3C). The NS5A self-interaction-enhancing H-38A mutant did not significantly reduce the NS5A-CypA interaction (Fig 7B), which correlates with the relatively moderate effect of this mutation on viral RNA replication (Fig 3C).

The results of NS5A dimer-interface mutations on the interaction between JFH1 NS5A and CypA could be summarized as follows. First, the J-36A mutant that showed the most significant defect in JFH1 NS5A self-interaction (Fig 5A) also had the most substantial defect in the JFH1 NS5A and CypA interaction (Fig 7C), which correlates nicely with the undetectable level of HJ3-5/36A RNA replication (Fig 5C). Second, both J-37A and J-38A did not show any significant effect on JFH NS5A-CypA interaction (Fig 7C), which also correlates with their relatively minor effects on NS5A self-interaction and viral RNA replication (Fig 5A and 5C). Third, despite having no impact on JFH1 NS5A self-interaction (Fig 5A), 112A mutation impaired the JFH1 NS5A-CypA interaction (Fig 7C), suggesting that the role of J112R on NS5A-CypA interaction could differ mechanistically from other residues involved in NS5A self-interaction. Fourth, the J-148A mutant showed a reduced NS5A-CypA interaction (Fig 7C), correlating with reduced J-148A self-interaction (Fig 5A) and impaired replication of the HJ3-5/148A mutant (Fig 5C).

In aggregate, these results suggest that NS5A self-interaction contributes to NS5A-CypA interaction, and that the impaired viral RNA replication observed from the majority of NS5A self-interaction-defective mutants could be due to a defective NS5A-CypA interaction.

### NS5A self-interaction regulates core and NS5A subcellular localization

**Fig 8:**
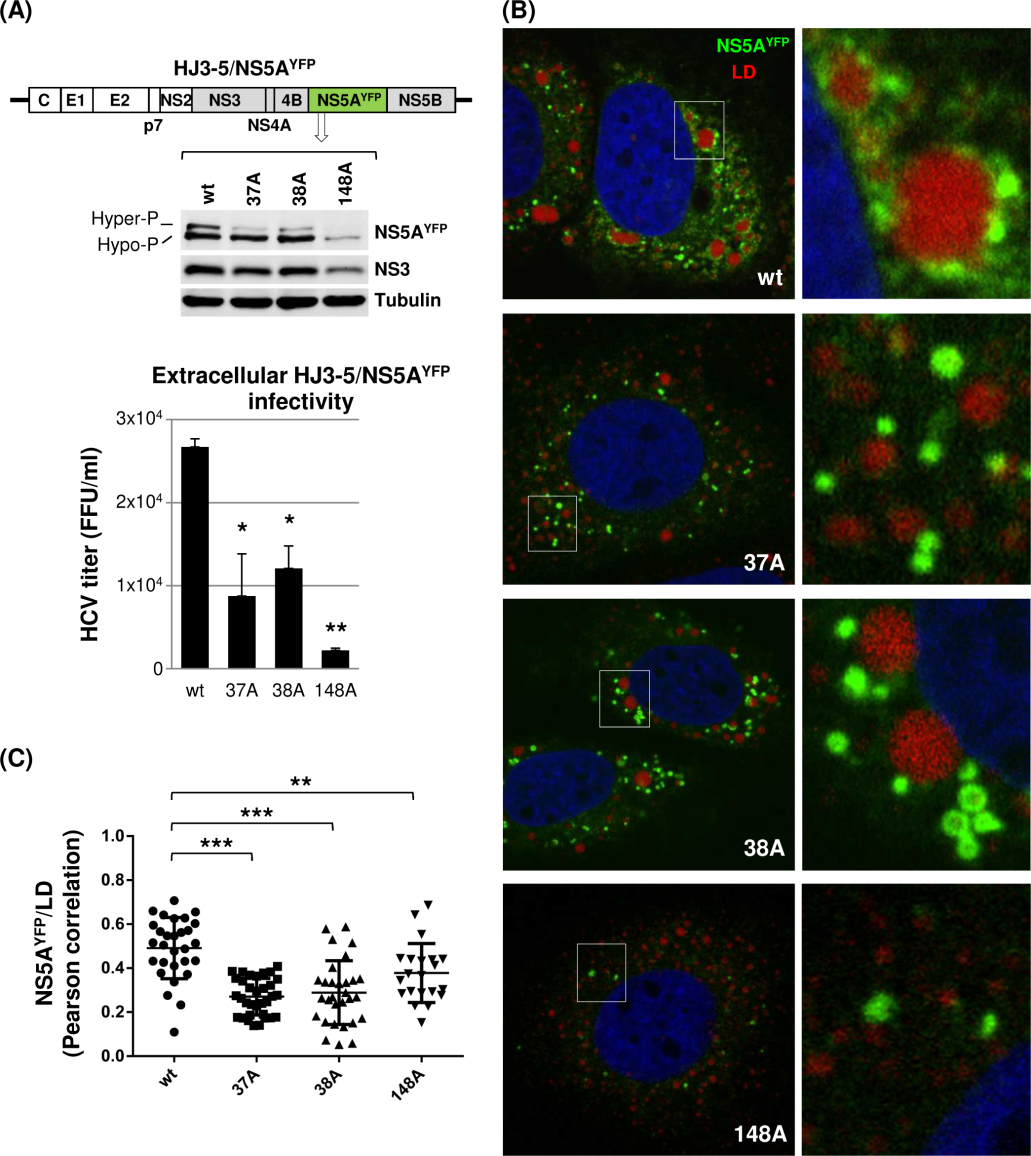
NS5A dimer interface mutations altered subcellular distribution of NS5A. **(**A) Diagram of HJ3-5 encoding YFP-fused NS5A with different mutations at the top, western blot of NS5A, NS3 and tubulin in the middle, and virus titers at the bottom. Western blot and virus titration were performed by using cell lysates collected on day 3 post-electroporation of HJ3-5/NS5A^YFP^. (B) Confocal imaging analysis by using an Olympus Fluoview FV 10i confocal microscope of cells at day 3 post-electroporation of HJ3-5/NS5A^YFP^ RNA with or without indicated individual mutations following LipidTOX deep red neutral lipid stain to detect the lipid droplets (LD). At the right is the enlarged area of the image on the left showing the different NS5A and LD interaction between wt and mutants. (C) NS5A^YFP^ and LD co-localization determined by Pearson correlation coefficient. Each dot represents a single cell.

Hyperphosphorylation of NS5A was shown to contribute to infectious HCV production by regulating NS5A recruitment to low-density membranes in the vicinity of lipid droplets (LD) and facilitating NS5A-core interaction [17]. Since NS5A hyperphosphorylation, as well as infectious virus production, were reduced in NS5A self-interaction mutants, we asked whether these phenotypes were caused by impaired NS5A subcellular localization and/or its interaction with core protein. To facilitate the detection of the NS5A in an immunofluorescence assay and the NS5A-core interaction in a co-immunoprecipitation assay, we used HJ3-5/NS5A^YFP^, which encodes YFP-tag within the NS5A-DIII and is capable of virus production (Fig 8A) [6]. First, we confirmed that NS5A dimer interface mutations in HJ3-5/NS5A^YFP^ also impaired NS5A hyperphosphorylation and virus production (Fig 8A). To determine the NS5A subcellular localization, Huh-7 cells electroporated with either wt HJ3-5/NS5A^YFP^ or its 37A, 38A and 148A mutants were subjected to confocal imaging analysis following a LipidTOX deep-red lipid staining to detect the LD. As shown in Fig 8B, we frequently detected a tight association between wt NS5A (measured by YFP fluorescence) and LD. However, in the case of NS5A mutants, a majority of NS5A was detected as the distinct foci in the cytoplasm without the tight LD association (Fig 8B). In fact, significantly lower degrees of NS5A-LD co-localization were calculated from the mutants compared to those from the wt, based on Pearson’s correlation measurements derived from the confocal images obtained from ~30 different cells (with the means of Pearson’s correlation coefficients for wt equaling 0.5206 versus 0.2598, 0.2424 and 0.3245 for 37A, 38A and 148A mutants, respectively) (Fig 8C).

**Fig 9:**
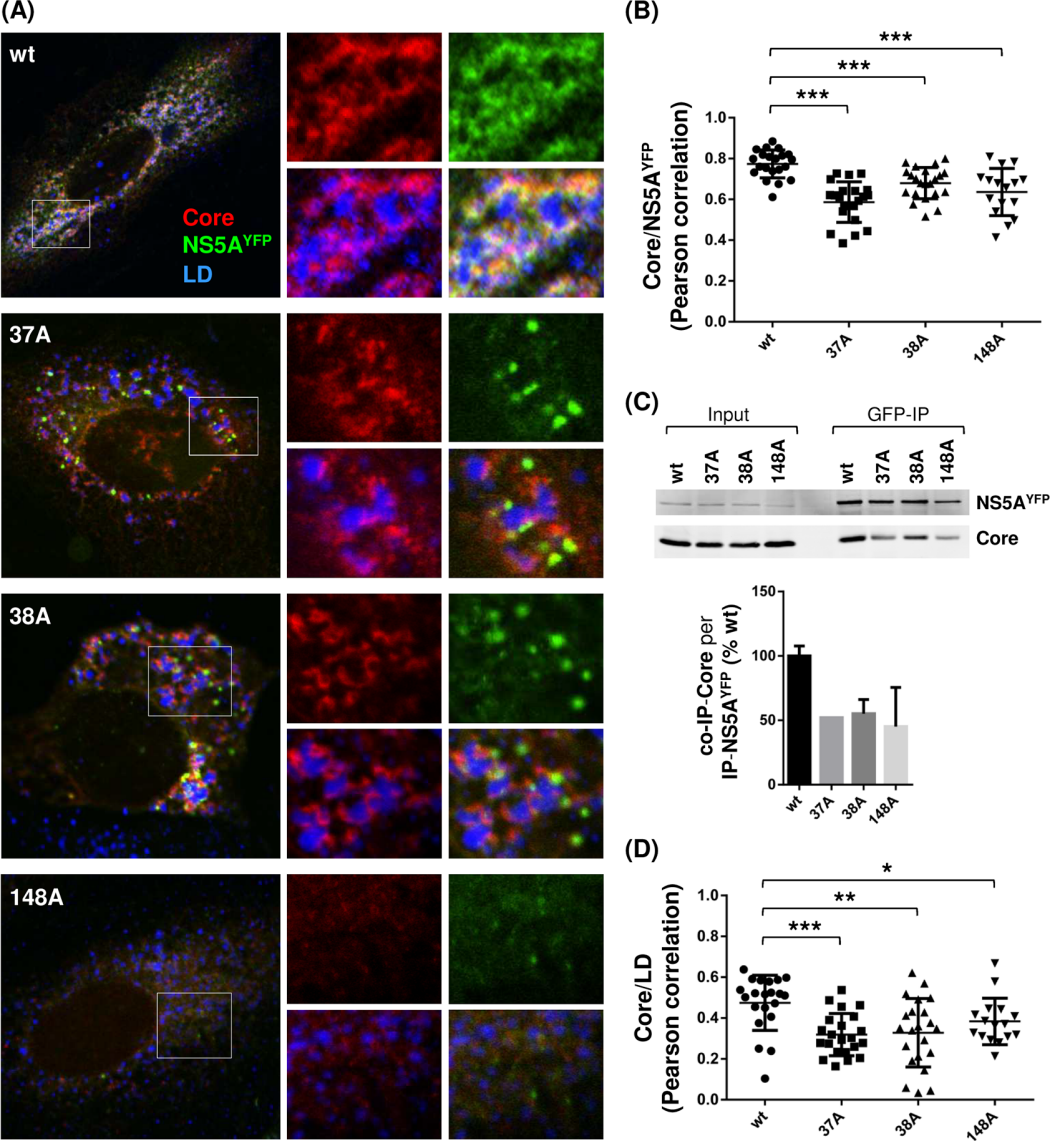
NS5A dimer interface mutations impaired NS5A and core interaction. **(**A) Confocal imaging analysis of cells at day 3 post-electroporation of HJ3-5/NS5A^YFP^ RNA with or without indicated individual mutations following core immunostaining (Alexa Fluor 405) and LipidTOX deep red neutral lipid stain. At the right is the enlarged area from individual channel or merged-images showing the core and NS5A localization relative to each other. (B) NS5A^YFP^ and core co-localization determined by Pearson correlation coefficient. (C) GFP immunoprecipitation (IP) followed by western blot analysis to detect NS5A and core interaction. Shown at the bottom is the relative NS5A and core co-immunoprecipitation (co-IP) efficiency from two independent experiments. (D) Core and LD co-localization determined by Pearson correlation coefficient.

The NS5A-core interaction was measured by two different methods: NS5A-core co-localization and co-immunoprecipitation (co-IP). NS5A-core co-localization was determined by performing confocal imaging analysis following immunostaining of core by using core-specific antibody in cells replicating HJ3-5/NS5A^YFP^ (Fig 9A). As shown in Fig 9B, a strong degree of co-localization was detected between wt NS5A and core (with a mean of Pearson’s correlation coefficient equaling 0.7726). However, lesser degrees of co-localization between these two proteins were detected from the 37A, 38A and 148A mutants (with the means of Pearson’s correlation coefficients equaling 0.5742 0.6564 and 0.6100, respectively) (Fig 9B). Next, we determined the NS5A-core interaction by performing a GFP-pull down assay. As shown in Fig 9C, compared to wt, NS5A mutants showed reduced NS5A and core co-IP efficiency (~50% lower than wt) expressed as a ratio of co-IP-core level per immunoprecipitated (IP)-NS5A^YFP^. These NS5A-core co-IP results correlate well with their co-localization data (Fig 9A and 9B) and indicate that NS5A-core interaction in NS5A mutants was reduced compared to that in wt. These results indicate that NS5A self-interaction regulates subcellular localization of NS5A and NS5A-core interaction. Since previous study showed the core-dependent recruitment of NS5A to LD-associated membranes [53], it is possible that NS5A self-interaction is critical for its interaction with core, which then promotes NS5A localization to LD-associated membranes. Alternatively, NS5A self-interaction promoted the NS5A localization to LD-associated membranes, consequently enhancing the interaction between NS5A and core at these membranes. Interestingly, all three NS5A mutants defective in self-interaction also reduced the core localization to the LD (Fig 9D). These results are consistent with recent study by Yin et al., which showed the reduced core localization to the LD by using other NS5A mutants (V67A or P145A), also defective in NS5A self-interaction [42].

### JFH1 NS5A/36V revertant rescued viral assembly and infectious virus production by restoring NS5A self-interaction and NS5A-CypA interaction

To better understand the role of NS5A self-interaction in HCV replication, we attempted to isolate revertants with primary- or second-site mutations that could rescue viral replication. This was done by continuously sub-culturing the Huh-7 cells electroporated with H77D or HJ3-5 encoding different NS5A interface mutations and monitoring the viral replication every 3 days. Among mutants that showed no evidence of transient replication, including 36A and 112A mutants in H77D or HJ3-5 background, only the HJ3-5/36A mutant showed strong evidence of viral replication and infectious virus production by day 10 post electroporation of this viral RNA to Huh-7 cells. Sequencing of the entire coding region of secreted HJ3-5/36A-derived revertant collected at 28 days post-electroporation cell culture supernatants reveled a single mutation in JFH-1 NS5A at residue position 36 to valine (36V). This was not a wt reversion since the wt JFH1 NS5A residue in this position is a phenylalanine.

**Fig 10:**
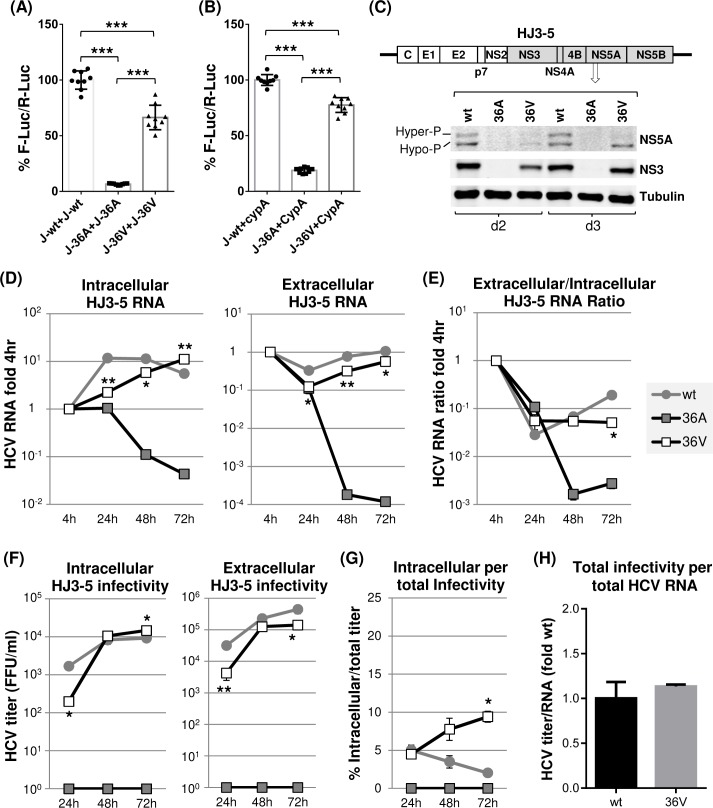
JFH1 NS5A/36V revertant partially restored most of the 36A mutation-mediated defects. Checkmate mammalian two-hybrid analyses were performed to determine the effect of 36V mutation on JFH1 NS5A self-interaction (A) and NS5A-CypA interaction (B). The effect of 36V mutation on NS5A hyperphosphorylation (C), HJ3-5 RNA replication (D-E) and infectious virus production (F-G) and the relative ratio of total infectivity per total HCV RNA levels (H) as described in detail in Figs 4 and 6.

Since, JFH1 NS5A/36A was defective in NS5A self-interaction (Fig 5A) and NS5A-CypA interaction (Fig 7C), which, we believe, has caused defective HJ3-5/36A replication (Fig 5B and 5C), we asked whether the 36V mutation is capable of restoring all of these defects associated with NS5A/36A mutation. To answer this question, we determined the effect of 36V mutation on the efficiency of JFH1 NS5A self-interaction and NS5A-CypA interaction by using checkmate assays as described above. As shown in Fig 10A and 10B, the 36V mutation in JFH1 NS5A significantly restored the 36A mutation-mediated impairments in NS5A self-interaction and NS5A-CypA interaction. Next, to verify that the 36V mutation in JFH1 NS5A was indeed responsible for the emergence of a replicable revertant from HJ3-5/36A mutant, we introduced the 36V mutation to HJ3-5. The results shown in Fig 10C to 10F indicate that HJ3-5/36V substantially restored viral RNA replication and virus production. HJ3-5/36V also showed reduced virus secretion compared to wt HJ3-5 (Fig 10E and 10G) without affecting virus assembly efficiency (Fig 10H). It is also notable that NS5A hyperphosphorylation in the HJ3-5/36V mutant was significantly lower than that in wt HJ3-5, probably due to only a partial restoration of NS5A self-interaction by a 36V mutation in NS5A (Fig 10A and 10C). In fact, all of these replication phenotypes of HJ3-5/36V revertant strikingly resemble those of HJ3-5/37A and HJ3-5/38A mutants. Overall, these results verified the concept that JFH1 NS5A self-interaction is critical for the NS5A-CypA interaction and NS5A hyperphosphorylation, which contribute to efficient HCV RNA replication and virus secretion.

**Fig 11:**
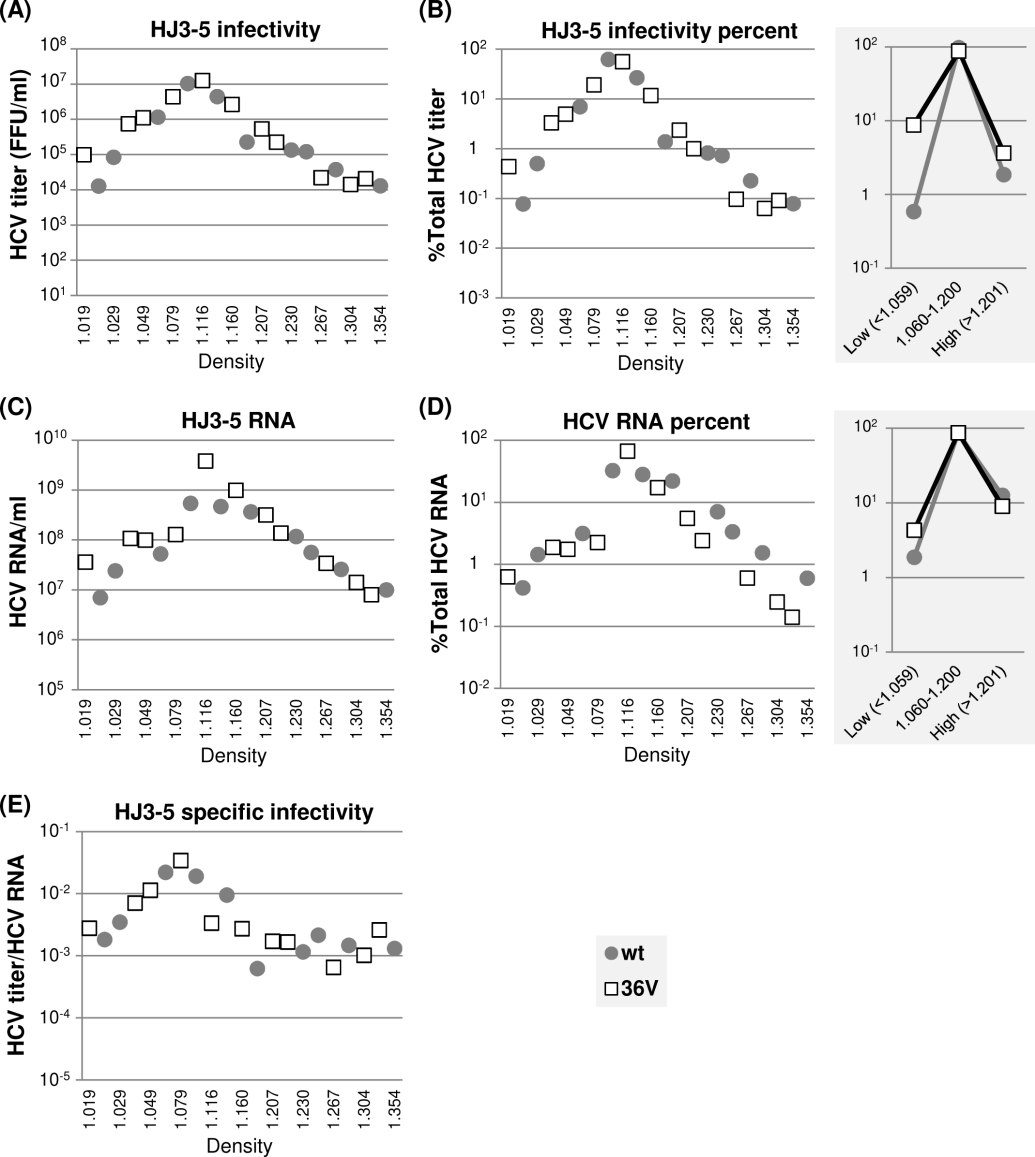
The effects of JFH1 NS5A/36V revertant on HJ3-5 virus particle density distribution. **(**A) Distribution of infectious virus present in different density fractions following equilibrium ultracentrifugation of cell culture supernatants collected between 48 h and 72 h post-electroporation of HJ3-5 and 36V mutants to Huh-7 cells. (B) The percentages of virus titers present at different density fractions relative to total titers from individual viruses are shown at left and the percentages of virus titers present in three different density ranges at right. (C) Distribution of HCV RNA at different density fractions as determined by qRT-PCR. (D) The percentages of HCV RNA levels present at different density fractions relative to total HCV RNA from HJ3-5 and HJ3-5/36V revertant are shown at left and the HCV RNA percentages present in three different density ranges at right. (E) The distribution of virus specific infectivity, calculated as the ratio of virus titer and HCV RNA, distribution at different density fractions.

Next, we determined the density and specific infectivity of extracellular HJ3-5/36V. Overall, density profiles of viral RNA and infectivity of this mutant closely resembled to other replication-competent, NS5A self-interaction defective NS5A mutants, including 37A, 38A and 148A mutants (compare Figs 6 and 11), reflecting the fact that 36V mutant is a partial revertant showing significantly lower NS5A self-interaction than wt (Fig 10A). Accordingly, similar to above three mutants, the relative titers of low-density (<1.059 g/cm^3^) 36V revertant were ~10 folds higher than those of the wt HJ3-5 (compare Figs 6B and 11B right panels). Due to this, the specific infectivity of 36V mutant viruses at low-density fractions was also higher than wt HJ3-5 (Fig 11E), similar to other mutants. Interestingly, however, proportions of 36V mutant RNA and infectivity, respectively, detected at high density fractions (>1.201 g/cm^3^) were much higher than those of other mutants, but comparable to those of wt HJ3-5 (compare Fig 6B and 6D and Fig 11B and 11D, right panels, see below and also Discussion).

**Fig 12:**
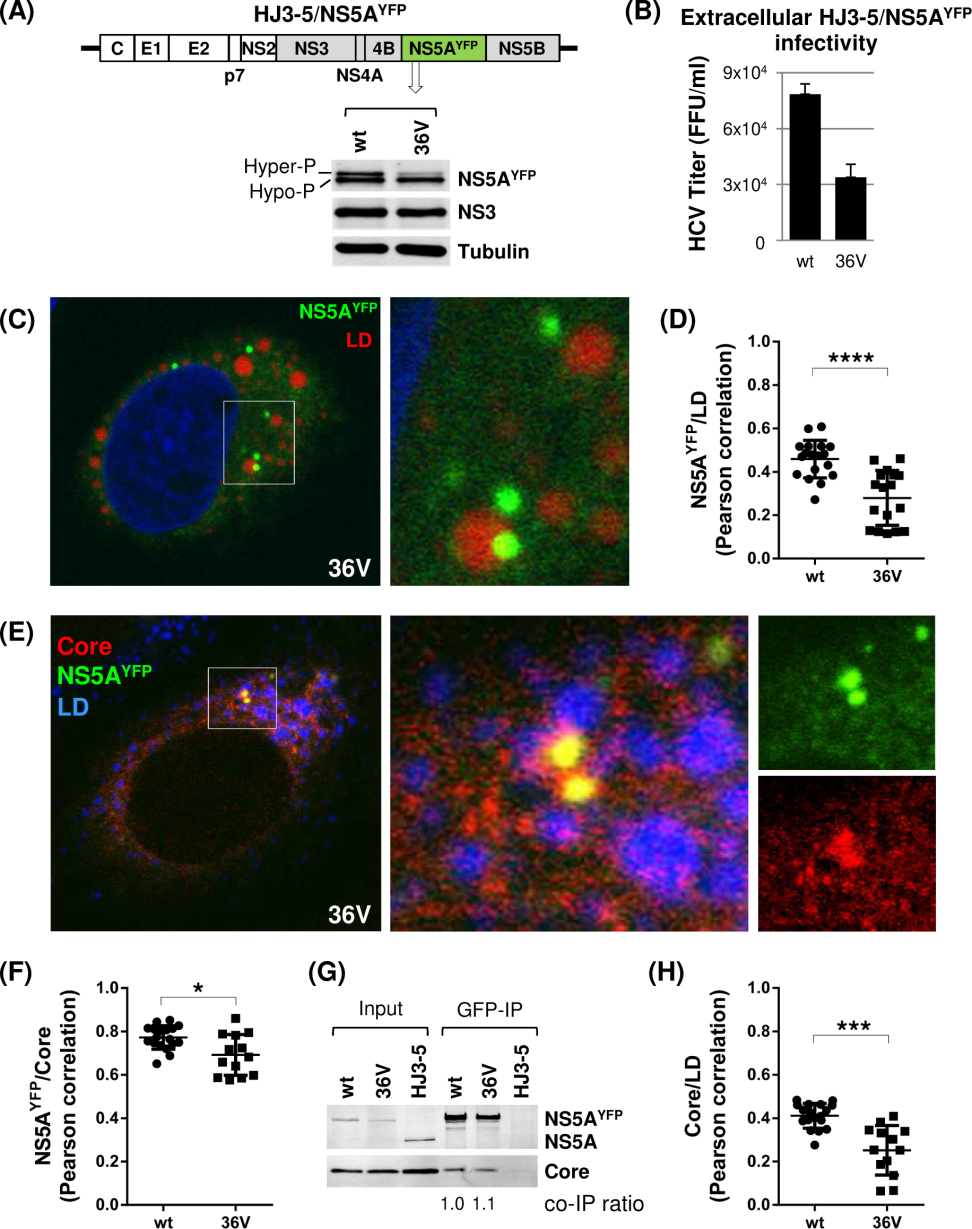
The 36V mutation altered NS5A subcellular localization and NS5A-core co-localization without affecting NS5A-core interaction affinity. **(**A) Diagram of HJ3-5 encoding YFP-fused NS5A with 36V mutation at the top, and Western blot of NS5A, NS3 and tubulin at the bottom. (B) Virus titers from HJ3-5/NS5A^YFP^ and 36V mutant are shown. Western blot and virus titration were performed by using cell lysates collected on day 3 post-electroporation of HJ3-5/NS5A^YFP^ and its 36V mutant. (C) Confocal imaging analysis by using an Olympus Fluoview FV 10i confocal microscope of cells at day 3 post-electroporation of HJ3-5/NS5A^YFP^/36V RNA following LipidTOX deep red neutral lipid stain to detect the lipid droplets (LD). At the right is the enlarged area of the image on the left showing the NS5A^YFP^/36V and LD interaction. (D) NS5A^YFP^/36V and LD co-localization determined by Pearson correlation coefficient. Each dot represents a single cell. **(**E) Confocal imaging analysis following core immunostaining (Alexa Fluor 405) and LipidTOX deep red neutral lipid stain. At the right is the enlarged area from individual channel or merged-images showing the core and NS5A^YFP^/36V. (F) NS5A^YFP^/36V and core co-localization determined by Pearson correlation coefficient. (G) GFP immunoprecipitation (IP) followed by western blot analysis to detect NS5A^YFP^/36V and core interaction by using cell lysates derived from HJ3-5/NS5A^YFP^ wt and 36V mutant replicating cells. The HJ3-5 encoding NS5A without GFP tag was used as the negative control of GFP-IP. Shown at the bottom is the relative NS5A and core co-immunoprecipitation (co-IP) efficiency. (H) Core and LD co-localization determined by Pearson correlation coefficient.

To determine the effect of 36V mutation on NS5A subcellular localization, we introduced this mutation to HJ3-5/NS5A^YFP^, and then confirmed that HJ3-5/NS5A^YFP^ /36V mutant is defective in NS5A hyperphosphorylation and virus production compared to wt, similar to the phenotypes of HJ3-5/36V (Fig 12A and 12B). Confocal imaging analysis revealed that a majority of NS5A^YFP^/36V was detected as the distinct foci in the cytoplasm without the tight LD association (Fig 12C), which is consistent with low degree of NS5A^YFP^ and LD co-localization based on Pearson’s correlation measurements (Fig 12D). Also we detected reduced degree of NS5A^YFP^ and core co-localization as well as core-LD association (Fig 12F and 12H). These NS5A^YFP^/36V phenotypes resembled those of other NS5A^YFP^ mutants shown in Figs 8 and 9, probably because they shared a common defect in NS5A hyperphosphorylation, which was shown to facilitate NS5A localization to the LD as well as NS5A and core co-localization [17]. However, uniquely to 36V mutant, we consistently detected small fraction of large NS5A foci, co-localizing with LD and core (Fig 12C and 12E). Also, we detected wt level pull-down of core by NS5A^YFP^/36V (Fig 12G), which, apparently, is contradictory to the reduced degree of NS5A^YFP^ and core co-localization detected from this mutant compared to wt (Fig 12F). Based on these data, we propose that 36V mutation in NS5A may have enhanced its affinity to core, partially compensating its LD localization defect caused by its defective hyperphosphorylation, resulting in fraction of NS5A recruitment to LD in core-NS5A interaction dependent manner. We speculate that LD-localized NS5A/36V may have contributed to near wt level of high-density particles detected from HJ3-5/36V mutant (Fig 11B and 11D, right panels). However, further study will be needed to verify this point.

## Discussion

HCV NS5A is a multifunctional protein involved in both viral RNA replication and virus production [24, 54, 55]. Our study provided mechanistic insights regarding the roles of, crystal structure-defined, NS5A dimer interface residues in these two critical steps in the HCV life cycle. Specifically, our data revealed that these residues regulate NS5A self-interaction, NS5A-CypA interaction, NS5A hyperphosphorylation, NS5A localization to LD and NS5A-core interaction, promoting HCV replication and infectious HCV production.

Among three domains of NS5A, DI plays a major role in NS5A self-interaction [39]. Currently three independent crystal structures of NS5A-DI, two from gt1b (Con1 strain) and one from gt1a (H77 strain), are available [36–38]. While their monomeric structures were similar to each other with an average Cα RMSD (root-mean-square deviation) equal or less than 1 Å [37, 38], four distinct dimeric forms were detected in different crystal packing conditions, including the two forms from gt1b shown in Fig 1 [36–38]. Interestingly, these NS5A dimeric forms are not mutually exclusive but have a potential to form NS5A oligomers via multiple different interfaces [37, 38]. Our data shown in Fig 2B support this possibility, since combining mutations located at two different interfaces additively reduced the level of NS5A self-interaction. Importantly, the fact that NS5A mutations located at different dimeric interfaces exhibited similar phenotypes, including their effects on NS5A hyperphosphorylation, NS5A-CypA interaction and NS5A subcellular localization, and, consequently, viral RNA replication and virus assembly/egress (Figs 3 to 9), strongly suggests the cooperative roles of different dimeric interactions within the same complexes. From a functional point of view, NS5A oligomerization is desirable for its role in promoting the formation of DMV [31, 32], which are sites of HCV RNA replication. In addition, the NS5A oligomerization model may be the best way to explain the high potency of NS5A inhibitors, which corresponds to one molecule of NS5A inhibitor impacting ~ 50,000 molecules of NS5A as in the daclatasvir example [56], and a synergistic activity of different NS5A inhibitors in re-sensitization of drug-resistant NS5A variants [56].

The structure of gt2a JFH1 NS5A is currently unknown. However, a substantial NS5A-DI sequence difference exists between gt2a JFH1 and gt1b Con1 (69% amino acid homology). Thus, it was remarkable that two-to-five mutations introduced to JFH1 NS5A-DI residues located at positions corresponding to two different gt1b NS5A-DI dimer-interfaces significantly inhibited its self-interaction at the levels similar to those of gt1a H77 NS5A-DI (compare Fig 2B and 2C), since gt1a H77 NS5A-DI is more homologous to gt1b NS5A-DI in sequence (82% amino acid homology) and structure (average Cα RMSD of 0.57 Å) [36–38]. Interestingly, the impacts of individual NS5A-DI dimer-interface mutations on NS5A self-interaction were different between H77 and JFH1-derived NS5A (Figs 3A and 5A). This difference was most apparent for R112A mutation, since this mutation severely impaired H77 NS5A self-interaction, while same mutation had no effect on JFH1 NS5A self-interaction. These results suggest that some intermolecular NS5A residue interactions might vary in these two HCV isolates due to differences in near neighbor residues. Supporting this interpretation, our preliminary data indicate that R112 residue in JFH1 NS5A may not participate in NS5A self-interaction, since neither (similarly charged) R112K nor (oppositely charged) R112E mutations affected this interaction (S2 Fig). On the contrary, JFH1 NS5A self-interaction was severely impaired by E148R mutation, but unaffected by E148D mutation (S2 Fig). These results suggest that E148 residue in JFH1 NS5A is involved in NS5A self-interaction via salt bridge formation, similar to E148 residue in H77 NS5A, but with different residue(s) instead of R112. However, JFH1 NS5A-DI structure determination will be necessary to identify the exact residues involved in NS5A self-interactions at its dimer interface(s).

The NS5A mutation-mediated impairment in NS5A self-interaction correlated with HCV RNA replication-defects driven by either H77 NS5A or JFH1 NS5A-containing viral replicases (Figs 3 and 5). These results suggest that NS5A self-interaction is critical for HCV RNA replication regardless of HCV genotypes. Interestingly, we detected a strong correlation between NS5A self-interaction and NS5A-CypA interaction that was shown to be critical for HCV RNA replication [52] (Figs 3, 5 and 7). Since the mutations we tested are located at NS5A-DI rather than at NS5A-DII that encodes the CypA interacting domain [57, 58], a direct role of these mutations in disrupting NS5A-CypA interaction is unlikely. Also, two mutations in NS5A (D316E/Y317N), which conferred the CypA-independent HCV replication phenotype [58], did not affect H77 NS5A self-interaction and slightly reduced JFH1-NS5A interaction (S3A Fig). These results support that NS5A self-interaction is driving NS5A-CypA interaction, and not vice versa. Interestingly, NS5A-CypA interaction was detected only from GAL4/BD-CypA and VP16/AD-NS5A pairs and not in reverse configuration (S4 Fig). We believe that these data support our interpretation that NS5A oligomers may interact with CypA, since we could easily envision a soluble VP16/AD-NS5A, not a DNA-bound GAL4BD-NS5A, forming a CypA-binding-competent oligomer. Now, how could NS5A self-interaction affect NS5A-CypA interaction? We propose that NS5A self-interaction may modulate the orientation of CypA-binding region in NS5A-DII, likely within the context of oligomeric NS5A structure, allowing efficient CypA binding.

Previous study by Lim et al. showed that mutating zinc-binding cysteines to alanine (C to A) within NS5A-DI (C39A, C57A, C59A and C80A) disrupted NS5A self-interaction and HCV RNA replication by using bacterially expressed and purified proteins [20]. These data support the role of NS5A self-interaction in HCV replication. However, NS5A-CypA interaction was not affected by any of eleven C to A mutations within full length NS5A (including the four zinc binding residues mentioned above), regardless of their impact on NS5A self-interaction [20]. These findings are different from our data, which showed positive correlation between NS5A self-interaction and NS5A-CypA interaction. We speculate that potential difference in NS5A oligomeric states between current and previous experimental systems might have altered the availability of CypA-interacting region in NS5A-DII for CypA binding leading to different outcomes.

Our data indicated that impaired gt1a H77 NS5A self-interaction resulted in virus particle assembly defects (Fig 3G), while that of gt2a JFH1 NS5A resulted in virus secretion defects (Fig 5F). Although this genotypic difference in regard to assembly versus secretion is difficult to understand, we could envision that more than 10 folds higher viral replication from HJ3-5 (encoding JFH1 NS5A), compared to H77D (encoding H77 NS5A), could have altered the relative roles of NS5A self-interactions in viral assembly/egress processes. Alternatively, H77 NS5A and JFH1 NS5A contributed to HCV assembly/egress via intrinsically different mechanisms. Regardless, this genotypic difference was not due to the chimeric nature of HJ3-5, which encode H77 core to NS2 in the background of JFH1, since NS5A dimer interface mutations introduced to full length JFH1/QL showed exactly same virus secretion defect as did HJ3-5 mutants (S5 Fig). Interestingly, while H77 NS5A mutants showed no effect on virus density or specific infectivity (Fig 4), JFH1 NS5A mutants increased the proportion of low-density, high-infectivity particles (Fig 6). It seems paradoxical that the specific infectivity of JFH1 NS5A mutants was higher than that of wt considering the reduced overall infectivity of mutants. However, it is possible that slowed viral egress in JFH1 NS5A mutants allowed their enhanced lipidation, resulting in low-density, highly infectious viruses, while this type of slow maturation of wt virus was relatively decreased due to efficient virus secretion (Fig 5D and 5F). Interestingly, LD localization of JFH1 NS5A dimer interface mutants was reduced, indicating that virus maturation into low-density particles may not strictly depend on a tight association of NS5A with the cytoplasmic LD. Alternatively, efficient LD localization of NS5A in wt HJ3-5-replicating cells could have enhanced virus secretion at a level to over-saturate the cells’ capacity for normal virus maturation, consequently, forcing the significant portion of viral particles to quickly egress as immature forms (Fig 6).

It is important to note that not all viral RNA secreted to the supernatant of HCV replicating cells is associated with infectious virus. In fact, Gastaminza et al. showed that HCV replicating cells released the low-density particles, including the exosome-like large vesicles, and high density particles, most likely representing non-enveloped core particles, in addition to majority of intermediate density particles corresponding to enveloped HCV particles [59]. Accordingly, low infectivity (relative to viral RNA) of both high- and low-density particles detected in our study could be attributed to these defective-particles, including exosomes and non-enveloped core particles. In this context, it is interesting to note that relative proportion of secreted, minimally infectious, high density viral particles from 36V revertant was significantly higher than those from 37A and 38A mutants (compare Figs 6 and 11), despite that most of other phenotypes of 36V revertant were similar to those of 37A and 38A mutants consistent with their similarly defective NS5A self-interaction. The second phenotypic difference between 36V revertant and these other mutants was the affinity of core and JFH1 NS5A, which was unchanged in the 36V revertant, but reduced in the 37A and 38A mutants, compared to that of wt (compare Figs 9C and 12G). Based on these data, we propose that high-affinity interaction between core and NS5A, independent from NS5A self-interaction, could promote the secretion of defective, high-density core particles.

Previously Miyanari et al. demonstrated that two different triple alanine mutations introduced to the residues 99–101 and 102–104 within NS5A-DI reduced NS5A localization to LD, establishing the role of NS5A-DI on NS5A localization to the LD [53]. Subsequently, Masaki et al. showed that NS5A hyperphosphorylation promote its LD localization [17]. Now, our data suggest that NS5A dimer-interface residues in NS5A-DI contribute to NS5A localization to the LD by regulating NS5A hyperphosphorylation (Figs 5 and 8). Interestingly, our extended study by using HCV polyprotein expression system indicate that all of NS5A dimer interface mutants that we tested, including 36A, 37A, 38A, 112A and 148A, regardless of their impact on NS5A self-interaction, could impair JFH1 NS5A hyperphosphorylation (S6 Fig). These data suggest that NS5A self-interaction per se may not be sufficient to promote its hyperphosphorylation. A recent study by Ross-Thriepland and Harris [23] indicated that JFH1 NS5A-DI residue 146 serine (146S) is a target of phosphorylation and mutating this residue to phosphomimetic aspartic acid (146D) led to decreased NS5A hyperphosphorylation. Since 146S is located near the 3FQM dimer interface in the vicinity of the 112R-148E salt-bridge, the authors predicted that phosphorylation at 146S might potentially regulate NS5A dimerization [23]. However, our data showed that 146A or 146D mutations did not significantly affect H77 or JFH1 NS5A self-interaction (S3B Fig). These data indicate that 146D mutation impaired NS5A hyperphosphorylation without affecting NS5A self-interaction similar to the phenotypes of 112A mutation (Fig 5A and S6 Fig). Consistent with these data, the NS5A inhibitors also reduced NS5A hyperphosphorylation [60], yet they did not affect NS5A self-interaction [40]. Intriguingly, NS5A inhibitors were implicated to affect intermolecular NS5A conformation [56, 61], and phosphorylation of NS5A residue 146 has a potential to alter local dimeric conformation [23]. Thus, these data may indicate that hyperphosphorylation of NS5A is dependent on its specific conformation that allows its interaction with kinases [such as casein kinase I-α (CKI-α)] involved in this process [17]. Accordingly, we propose that only a defined conformation of NS5A, mostly likely within the oligomeric complexes, permits the access of kinases to NS5A LCSI domain resulting NS5A hyperphosphorylation [17, 62], and disturbing the kinase-accessible conformation of NS5A either by different NS5A mutations or treatment with NS5A inhibitors impairs NS5A hyperphosphorylation.

It is possible that all or some of our mutants may have impacted HCV RNA replication and virus assembly by altering NS5A conformation or other functions of NS5A, in addition to altering NS5A self-interaction-mediated functions. However, the ultimate proof supporting the role of NS5A self-interaction in HCV RNA replication and virus production was provided by a 36 valine (36V) revertant mutation in JFH1 NS5A, which replaced the original alanine mutation that conferred a severe defect in JFH1 NS5A self-interaction. This JFH1 NS5A/36V mutation, not only significantly restored the NS5A self-interaction, but also restored NS5A-CypA interaction, and HJ3-5/36V RNA replication and infectious virus production. Interestingly, 36V mutation introduced to H77 NS5A also partially enhanced NS5A self-interaction as well as an NS5A-CypA interaction (S7 Fig). These data support the notion that 36V mutation in JFH1 NS5A was indeed selected to rescue the NS5A self-interaction-defect caused by 36A mutation. However, the H77D/36V mutant did not show detectable level of viral RNA replication. The exact reason for impaired replication of H77D/36V is unclear. However, it is interesting to note that H77 NS5A residue F36 points toward lipid phase in the 1ZH1 structure (Fig 1A), suggesting that F36 may contribute to NS5A and membrane interaction. Based on this, we speculate that 36V mutation may have failed to restore the F36’s additional function at the NS5A-membrane interface and, as a consequence, could not rescue H77D replication. This potential, additional role of F36 residue in H77D replication may also explain the reason H77D/36A mutant could not replicate when 148A mutant showed low level replication (Fig 3C), despite their similar NS5A self-interaction defects (Fig 3A).

**Fig 13:**
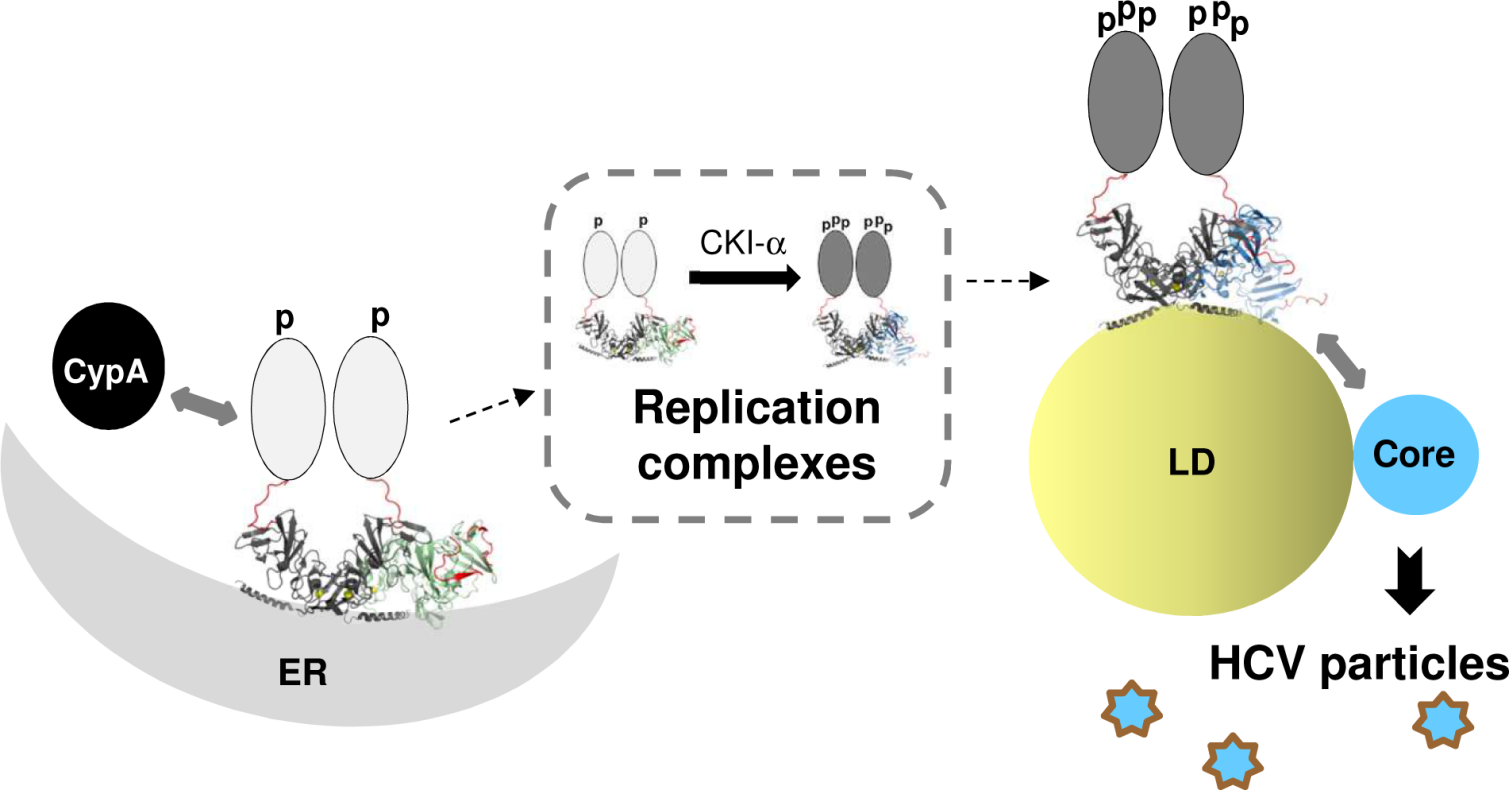
A model of functional role of NS5A oligomers during HCV replication. Oligomerization of NS5A may stabilize certain NS5A conformation allowing its interaction with CypA, which, together with NS5A oligomers, promote HCV replication complex (RC) formation. Interaction between NS5A and other HCV nonstructural proteins in the RC should then allow the access of kinases to hyperphosphorylate NS5A, which promotes NS5A association with LD and permits its interaction with core leading to infectious HCV production. See the discussion for detail. Hypothetical tetrameric arrangement of two heteromeric dimer forms of NS5A-DI (PDB-1ZH1 and PDB-3FQM) binding to negative curvature of ER at the left side and that of two homodimers (from PDB-1ZH1) binding to positive curvature of LD surface at the right are shown. The C-terminal tail portion is highlighted in red color in the structure to show the location and orientation of this region relative to the membrane surface. Artificial oval drawing representing the unstructured NS5A-DII and DIII were placed only on one of two dimers. The “p” and “ppp” represent hypo- and hyper-phosphorylated NS5A.

As illustrated in Fig 13, we propose that high-order NS5A oligomerization stabilizes NS5A conformation at local domains, including the DII and LCSI, which would allow them to interact with host proteins including CypA [63, 64] and CKI-α [17, 65]. The CypA-induced modulation of NS5A would then promote DMV formation, which will harbor HCV replication complexes [19, 30–32]. This early event will be followed by NS5A hyperphosphorylation by CKI-α [17, 22, 66], which would be promoted by interaction between NS5A and other HCV nonstructural proteins in the replication complexes as demonstrated in previous reports [67, 68]. Then the hyperphosphorylated NS5A (in the replication complexes) will move to low-density membrane domains near LD [53], interact with core protein, and promote HCV assembly and/or virus egress [17]. A recent pulse-chase imaging study by Wang and Tai strongly supports this scenario, since they determined that the NS5A-associated organelles (replication complexes) are continuously generated *de novo* and NS5A in the aged version of these organelles tend to associate with LD, accompanied with increases in NS5A hyperphosphorylation [66].

In conclusion, our study provided novel insights indicating that NS5A may function as oligomers formed via multiple dimeric interfaces to promote HCV RNA replication and virus production. It is likely that NS5A functions requiring its oligomerization make it an excellent target of highly potent inhibitors [29]. Although NS5A inhibitors did not perturb NS5A dimerization per se [20, 40, 69], it is suggested that they might have modulated its conformation [70] or higher-order NS5A oligomerization state [56]. In the future, understanding the detailed mechanistic function of NS5A oligomers during HCV replication, combined with determining the exact mode of action of NS5A inhibitors, will provide insights into understanding HCV replication mechanisms and for improving/identifying potent antivirals against HCV and other agents that utilize proteins functioning in an equivalent manner.

## Methods

### Plasmids

The construction of H77D and HJ3-5 was described previously [44, 48]. To generate the pairs of pACT and pBind vectors expressing full-length NS5A from two different genotypes, H77 and JFH1 NS5A sequences were PCR amplified from H77D and HJ3-5 with the primer sets introducing Sgf*I* and Pme*I* restriction enzyme sites at their N- and C-terminus, respectively, and then cloned into pFN10A(ACT) Flexi vector and pFN11A(BIND) Flexi Vector digested with Sgf*I* and Pme*I* enzymes (Promega, WI, USA). NS5A mutations were introduced by using the QuikChange II XL site-directed mutagenesis kit (Agilent Technology, Santa Clara, CA). The sequences of regions manipulated within each plasmid were verified by DNA sequencing. Other plasmids used for vector control (pACT and pBind), positive controls (pACT-MyoD and pBind-ID) and pGL4.3[*luc2P*/*Gal4*UAS/Hygro] were provided via a Checkmate/Flexi Mammalian Two-Hybrid System (Promega, WI, USA).

### Cell culture

Huh-7 cell lines used in this study including Huh7.5 (a clonal cell line of Huh-7, kindly provided by Dr. Charles M. Rice at Rockefeller University [71]) and FT3-7 (a clonal cell line of Huh-7 as described in [72]) were maintained in Dulbecco's modified Eagle medium (DMEM) (Invitrogen, Carlsbad, CA) containing 10% fetal bovine serum (Invitrogen, Carlsbad, CA) at 37°C in a 5% CO_2_ atmosphere.

### Mammalian two-hybrid assay

The interactions between NS5A-NS5A or NS5A-CypA were evaluated by using a Checkmate Mammalian Two-Hybrid System (Promega, WI, USA). In brief, pACT and pBIND based plasmids along with the pGL4.3[*luc2P*/*Gal4*UAS/Hygro] reporter plasmid were co-transfected into FT3-7 cells by using TransIT-LT1 (Mirus, Madison, WI) reagent according to the manufacturer’s instructions at a ratio of 3 μl transfection reagent per 1 μg of plasmid DNA. At 48 h post transfection, cell lysates were prepared to assess the firefly and Renilla luciferase activities by using a Dual-Luciferase Reporter (DLR) Assay System (Promega, WI, USA) and GloMax DISCOVER instrument (Promega, WI, USA) according to the manufacturer’s instructions.

### In vitro HCV RNA transcription and electroporation

HCV RNA was transcribed *in vitro* from linearized HCV cDNA by using the T7 MEGAscript Kit (Life Technologies, Carlsbad, CA) and purified by using an RNeasy RNA isolation kit (Qiagen, Valencia, CA). In brief, 5 x 10^6^ FT3-7 cells were mixed with 10 μg of HCV RNA in a 4-mm cuvette and pulsed once at 270 V and 950 μF by using a Gene Pulser System (Bio-Rad, Hercules, CA). Electroporated cells were transferred into 12-well plates for HCV RNA analysis and or 6-well plates or 6 cm dishes for virus titration and protein analysis.

### HCV titration

Extracellular and intracellular HCV titers in clarified cell culture supernatants and 4-cycle, freeze-thaw cell lysates harvested from FT3-7 cells at different time points post-electroporation of HCV RNA were determined by performing an HCV core antigen immunfluorescence assay as described before [73].

### Quantitative real-time RT-PCR assay

HCV RNA in cell culture supernatants and gradient fractions (see below-density gradient ultracentrifugation) was harvested by using a QIAamp viral RNA mini kit (Qiagen, Valencia, CA). Cell-associated HCV RNA was harvested by using an RNeasy RNA isolation kit (Qiagen, Valencia, CA). To quantitate the level of HCV RNA, a real-time RT-PCR assay was performed by using a QuantiNova Probe RT-PCR Kit (Qiagen, Valencia, CA) and a CFX96 real-time system (Bio-Rad, Hercules, CA) with custom designed primer probe sets (Sense primer: HCV84FP, 5’-GCCATGGCGTTAGTATGAGTGT-3’; antisense primer: HCV 303RP, 5’-CGCCCTATCAGGCAGTACCACAA-3’; and probe: HCV146BHQ, FAM-TCTGCGGAACCGGTGAGTACACC-DBH1). Briefly, 10 μl 2x QuantiNova Probe RT-PCR Master Mix, 1 μl each of 20 μM sense- and antisense primers, 0.16 μl of 20 μM HCV-specific probe, 0.2 μl of 100x QuantiNova Probe RT Mix, 4 μl of template RNA and RNase-free water were combined to make 20 μl reaction mixtures. HCV RNA was reverse transcribed for 10 min at 45°C followed by 5 min incubation at 95°C to activate PCR polymerase, then PCR was performed for 30 cycles of 95°C for 5 seconds (denaturation) and 60°C for 30 seconds (annealing and extension).

### Western blot analysis

Cell lysates were prepared in 1% CHAPS in PBS lysis buffer containing 1x protease- and phosphatase inhibitor cocktail mix (GenDEPOT, Katy, TX), separated by SDS-PAGE and transferred onto PVDF membranes. The membrane was blocked and probed with primary antibodies to core protein (1:2,000 dilution of C7-50, Thermo Scientific, Rockford, IL), NS3 (1:2,000 dilution of 9-G2, ViroGen, Watertown, MA), NS5A [1:15,000 dilution of 9E10 (kindly provided by Dr. Charles M. Rice at Rockefeller University) or 1:2000 dilution of 2F6, BioFront Technologies, Tallahassee, FL], and NS5A^YFP^ (1: 2000 dilution of anti-GFP, Life Technologies) and tubulin (1:7000 dilution, EMD Millipore, Billerica, MA). Protein bands were visualized by incubating the membranes with IRDye Secondary antibodies (Li-Cor Biosciences, Lincoln, NE), followed by imaging with an Odyssey infrared imaging system (Li-Cor Biosciences, Lincoln, NE).

### Virus concentration and density gradient ultracentrifugation

Approximately 1.5 x 10^7^ FT3-7 cells were electroporated with *in vitro*-transcribed RNAs and seeded into 175cm^2^ flasks. The HCV containing cull culture supernatants were collected from 48 to 72 h for every 4–6 h, pooled and centrifuged to remove cell debris. The clarified supernatants were loaded onto Centricon Plus-70 (Millipore, Germany), concentrated by centrifugation at 3,500 x g at 4°C and subjected to discontinuous Optiprep gradient centrifugation (60, 45, 30, and 15%) for 16h at 120,000 x g at 4°C in a SW55Ti rotor (Beckman, Indianapolis, IN). Each of 450 μl fraction was collected by aspiration from the top of the gradient and analyzed to determine its density, infectivity and amounts of HCV RNA as described above (see also [12]).

### GFP Co-immunoprecipitation

Cell lysates were prepared in 1 ml of lysis buffer [0.5% Triton X-100, 10mM Tris-HCl (pH 7.5), 150mM NaCl] containing 1x protease- and phosphatase inhibitor cocktail mix (GenDEPOT, Katy, TX) and incubated on ice for 1h. Cell lysates were incubated with anti-GFP magnetic beads (Miltenyi Biotech, Auburn, CA) for 1h at 4°C with gentle mixing and applied to μ columns. Magnetic beads were washed 4 times each with lysis buffer, and wash buffer I (150mM NaCl,1%NP-40, 0.5% sodium deoxycholate, 0.1% SDS, 50mM Tris-HCl, pH 8.0), respectively, followed by one wash with buffer 2 (20mM Tris-HCl, pH 7.5). Bound immune complexes were eluted from columns by applying a preheated SDS sample buffer.

### Confocal microscopy

HCV RNA electroporated cells were plated on 8-well chamber slides (BD Bioscience, Bedford, MA) at a density of 1x10^4^ cells per well. Two to three days later, the slides were washed with PBS, fixed with 4% formaldehyde for 20 min at room temperature, and permeabilized with 0.2% Triton X-100 in PBS for 10 min, then incubated overnight at 4 ^o^C with anti-core monoclonal antibody (1:2,000 dilution of C7-50, Thermo Scientific, Rockford, IL), followed by Alexa Fluor 405-conjugated goat anti-mouse antibody (1:1000 dilution, Invitrogen, Carlsbad, CA) for 1 h. Lipid droplets were stained with HCS LipidTOX deep red neutral lipid stain (1:1000 dilution, Molecular Probes Inc, Eugene, OR). The slides were examined with an Olympus FluoView FV10i confocal microscope (Olympus America Inc, Waltham, MA). Pearson’s coefficient was obtained by using FV10i-ASW 4.2 viewer software.

### Statistical analysis

Student’s t-test (unpaired) was performed by using GraphPad Prism version 6 software to determine the significance in differences between paired values. A P value less than 0.05 was considered statistically significant.

## Supporting information

S1 Fig The residue interaction network showing the non-covalent residue interactions within two different NS5A-DI dimer structures. We used experimental structure information from the Protein Databank RCSB PDB [74] for 3D structure analysis and computed residue-interaction networks (RINs) for PDB-1ZH1 **(A)** and PDB-3FQM **(B)** by using the RINerator tool [75]. To this end, hydrogens are first added to the 3D protein structure by using the Reduce tool [76]. Then contacts on the van-der-Waals (vdW) surface of each atom are sampled by the Probe tool [77] and are finally summarized into residue interactions by RINerator. In the resulting network, nodes represent protein residues and edges indicate non-covalent residue interactions between these residues. The following interaction types are identified: van-der-Waals contacts (cnt), hydrogen bonds (hbond), overlaps of van-der-Waals radii (ovl), and combined (any of the previous three). We also distinguish between two types of interacting atoms: main chain (mc) and side chain (sc). The RINs are loaded and visualized in Cytoscape (http://www.cytoscape.org) by using the RINalyzer and structureViz2 apps [78]. Residues from different NS5A monomers are colored in yellow and salmon and designated as “A” and “B”, respectively.
(TIF)

S2 Fig The effects of 112E, 122K, 148D and 148R mutations on JFH1 NS5A self-interaction. JFH1 NS5A self-interaction was determined by a checkmate mammalian two-hybrid assay. Statistical analysis was performed by using student’s t-test as described in Materials and Methods.
(TIF)

S3 Fig The effects of DEYN, 146A or 146D NS5A mutations on H77 or JFH1 NS5A self-interaction. The effects of NS5A-DEYN (D316E/Y317N) mutations **(A)** or 146A and 146D mutations **(B)** on H77 or JFH1 NS5A self-interaction as determined by a checkmate mammalian two-hybrid assay.
(TIF)

S4 Fig Detection of NS5A-CypA interaction only in GAL4/BD-CypA and VP16/AD-NS5A pair configuration of checkmate assay. CypA and NS5A interaction was determined by a checkmate mammalian two-hybrid assay. Statistical analysis was performed by using student’s t-test as described in Materials and Methods.
(TIF)

S5 Fig Effects of NS5A dimer interface mutations in JFH1/QL replication and infectivity. JFH1/QL was generated by introducing cell culture adaptive mutation in NS3 residue Q221L [44]. **(A)** NS5A western blot analysis of JFH1/QL wt and NS5A dimer interface mutants, which indicates the defective replication of 36A and 112A mutants and also the hyperphosphorylation impairment in 37A, 38A and 148A mutants. **(B)** Left and right panels show the virus titration results by using cell lysates (intracellular) and cell culture supernatants (extracellular), respectively, at different time points post-electroporation of JFH1/QL RNA with or without indicated mutations. **(C)** Percentage of intracellular virus titers per total (intracellular plus extracellular) virus titer.
(TIF)

S6 Fig JFH1 NS5A hyperphosphorylation and NS5A-core interaction detected following expression of HCV polyproteins. **(A)** Western blot analysis of NS5A^YFP^ following expression of full length HCV polyprotein derived from HJ3-5/NS5A^YFP^ by using T7 based expression system composed of pTM1 vector ([79], kindly provided by Dr. Bernard Moss at the National Institute of Health, USA) and T7-Lunet cells ([80], kindly provided by Dr. Bartenschlager at the University of Heidelberg, Germany). **(B)** GFP immunoprecipitation (IP) followed by western blot analysis to detect NS5A and core interaction. Shown at the bottom is the relative NS5A and core co-immunoprecipitation (co-IP) efficiency.
(TIF)

S7 Fig NS5A/36V mutations on H77 NS5A self-interaction and NS5A-CypA interaction. The effects of NS5A/36V mutations on H77 NS5A self-interaction **(A)** and NS5A-CypA interaction **(B)** as determined by a checkmate mammalian two-hybrid assay.
(TIF)
