# Supplementary figures and images for: HCV NS5A dimer interface residues regulate HCV replication by controlling its self-interaction, hyperphosphorylation, subcellular localization and interaction with cyclophilin A
Source: PLoS Pathog. 2018 Jul 23;14(7):e1007177. doi: 10.1371/journal.ppat.1007177 (PMC6072203; doi:10.1371/journal.ppat.1007177)

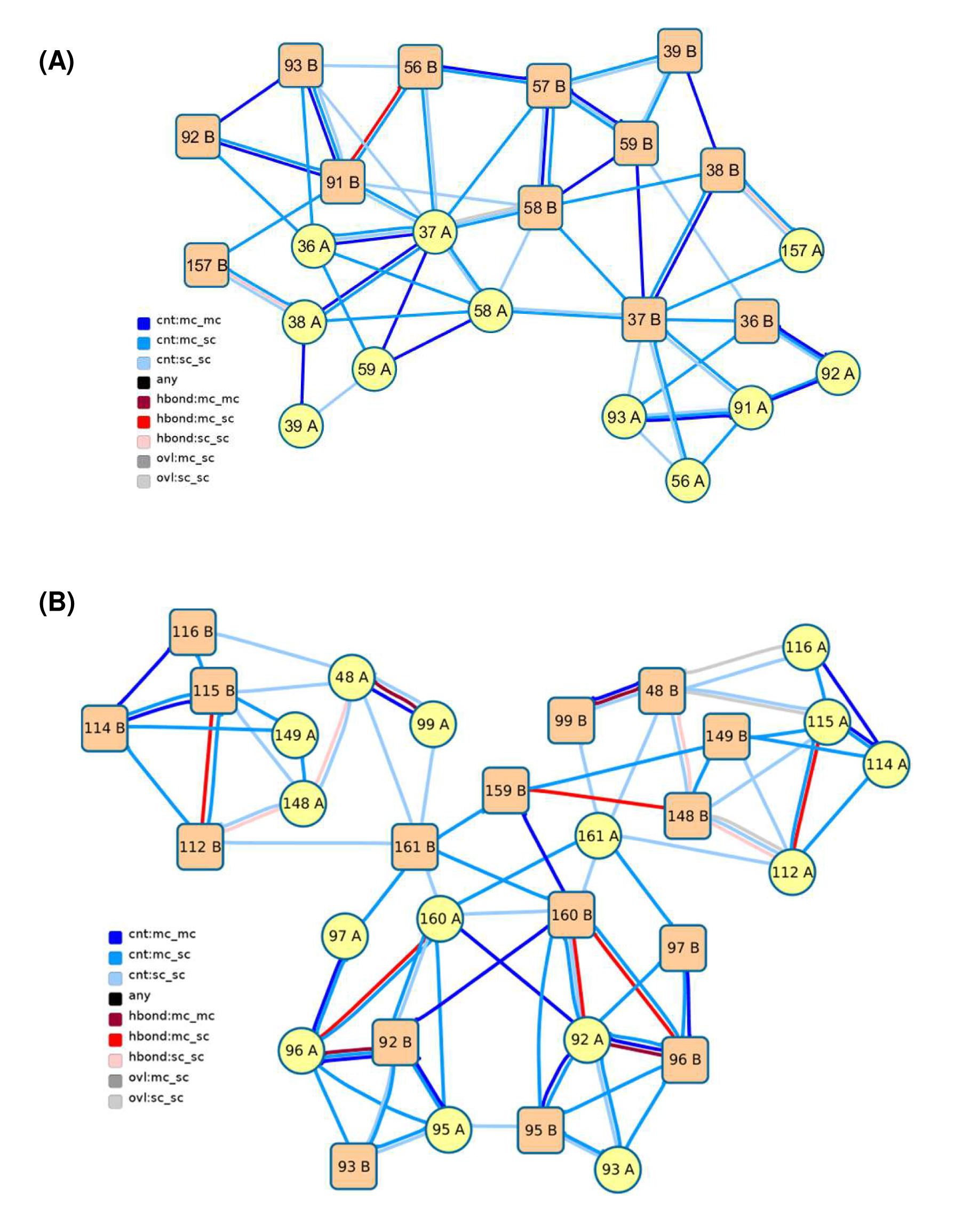

Supplement: S1 Fig — We used experimental structure information from the Protein Databank RCSB PDB [74] for 3D structure analysis and computed residue-interaction networks (RINs) for PDB-1ZH1 (A) and PDB-3FQM (B) by using the RINerator tool [75]. To this end, hydrogens are first added to the 3D protein structure by using the Reduce tool [76]. Then contacts on the van-der-Waals (vdW) surface of each atom are sampled by the Probe tool [77] and are finally summarized into residue interactions by RINerator. In the resulting network, nodes represent protein residues and edges indicate non-covalent residue interactions between these residues. The following interaction types are identified: van-der-Waals contacts (cnt), hydrogen bonds (hbond), overlaps of van-der-Waals radii (ovl), and combined (any of the previous three). We also distinguish between two types of interacting atoms: main chain (mc) and side chain (sc). The RINs are loaded and visualized in Cytoscape (http://www.cytoscape.org) by using the RINalyzer and structureViz2 apps [78]. Residues from different NS5A monomers are colored in yellow and salmon and designated as “A” and “B”, respectively. (TIF) [file ppat.1007177.s001.tif]

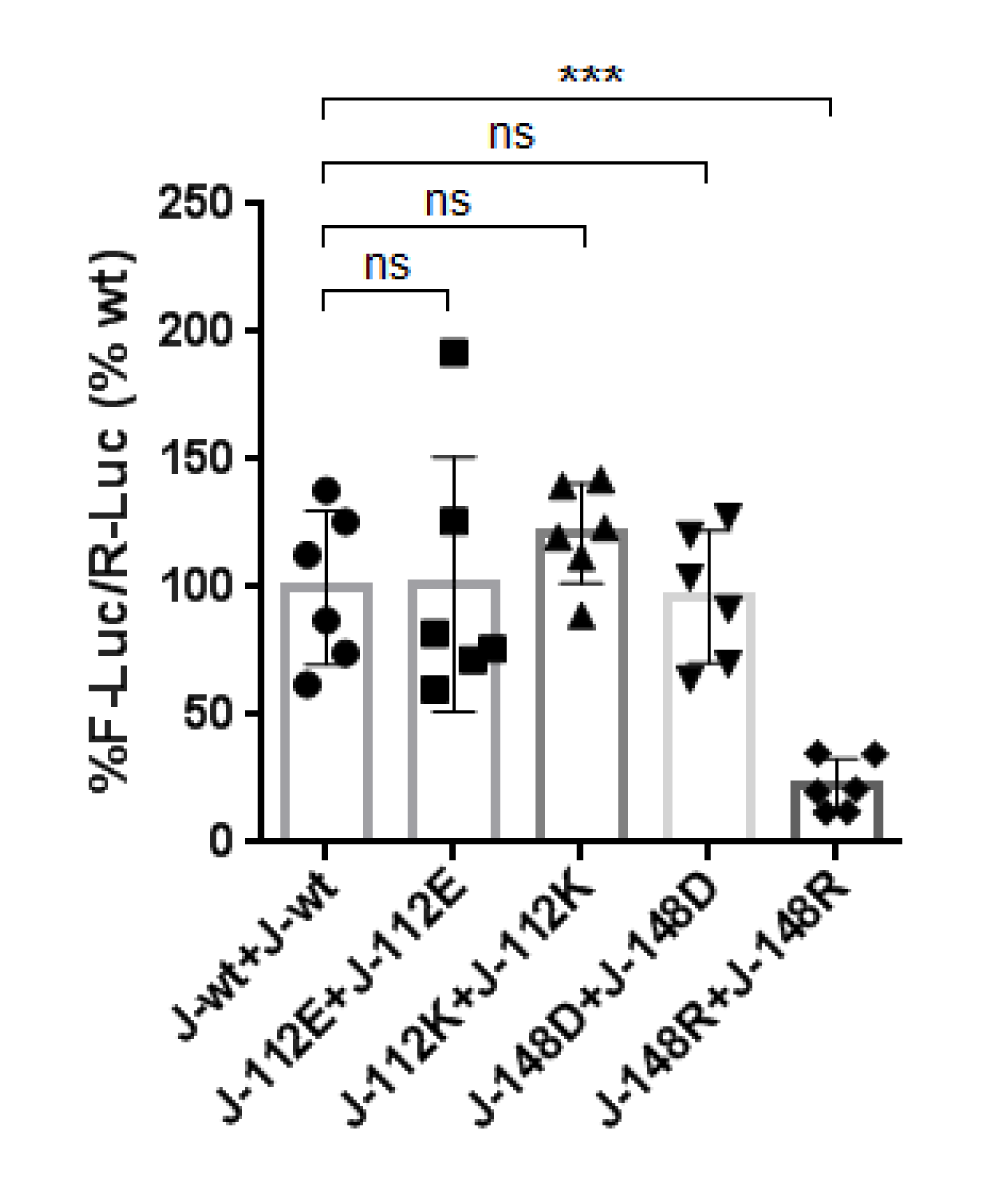

Supplement: S2 Fig — JFH1 NS5A self-interaction was determined by a checkmate mammalian two-hybrid assay. Statistical analysis was performed by using student’s t-test as described in Materials and Methods. (TIF) [file ppat.1007177.s002.tif]

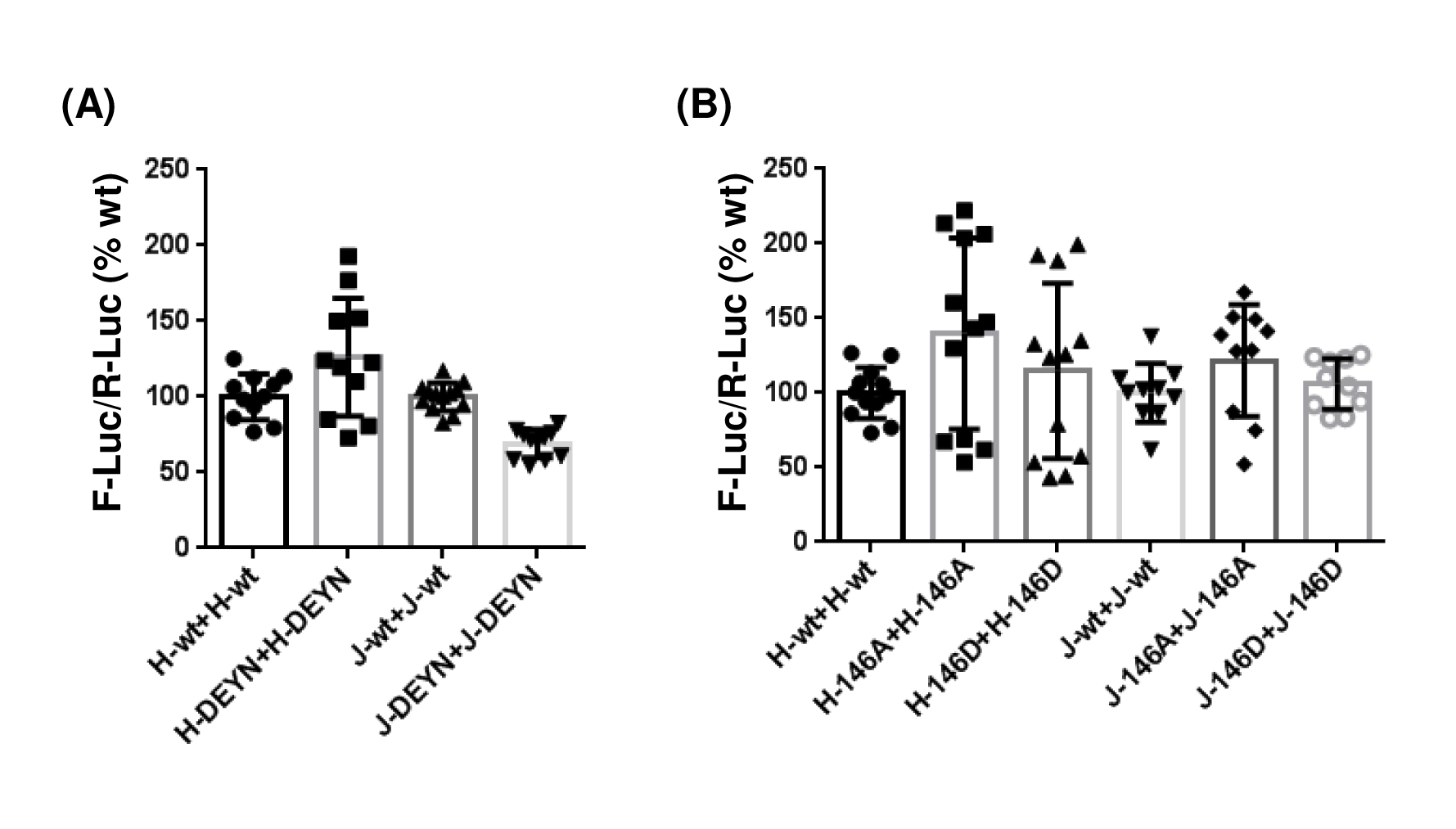

Supplement: S3 Fig — The effects of NS5A-DEYN (D316E/Y317N) mutations (A) or 146A and 146D mutations (B) on H77 or JFH1 NS5A self-interaction as determined by a checkmate mammalian two-hybrid assay. (TIF) [file ppat.1007177.s003.tif]

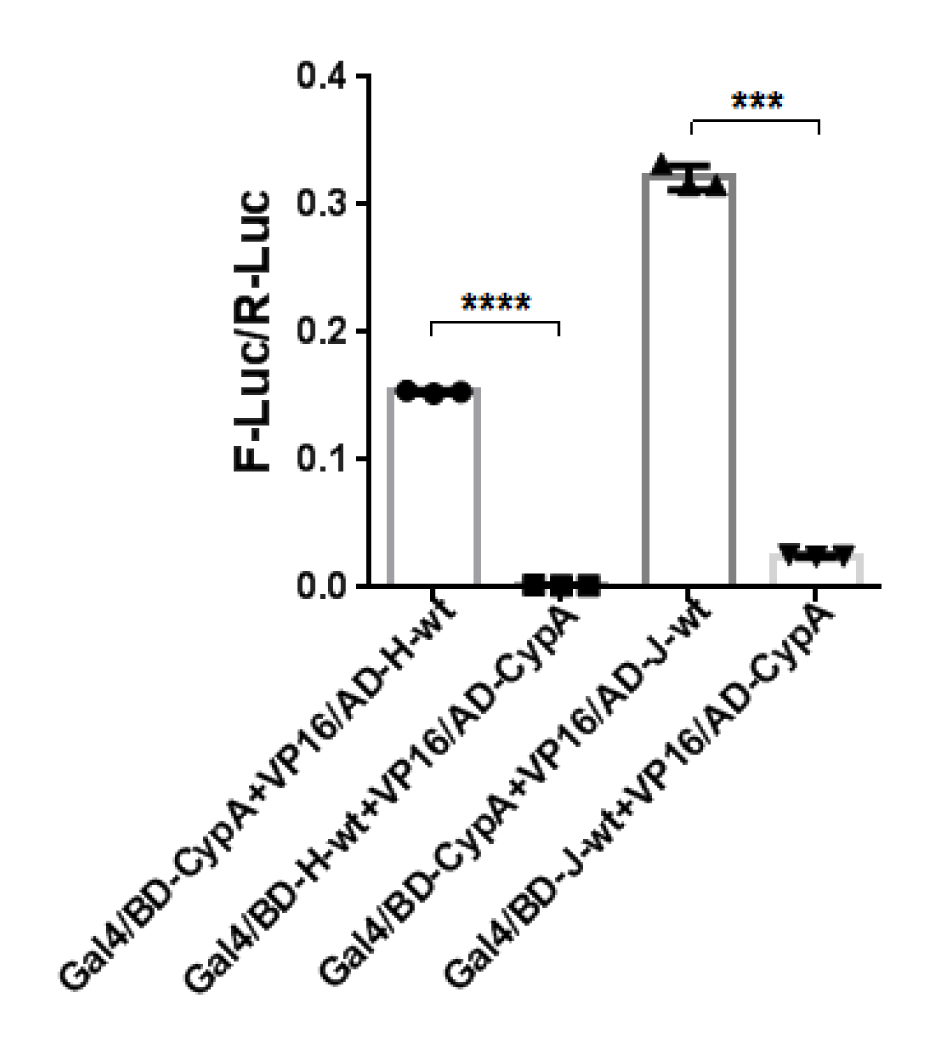

Supplement: S4 Fig — CypA and NS5A interaction was determined by a checkmate mammalian two-hybrid assay. Statistical analysis was performed by using student’s t-test as described in Materials and Methods. (TIF) [file ppat.1007177.s004.tif]

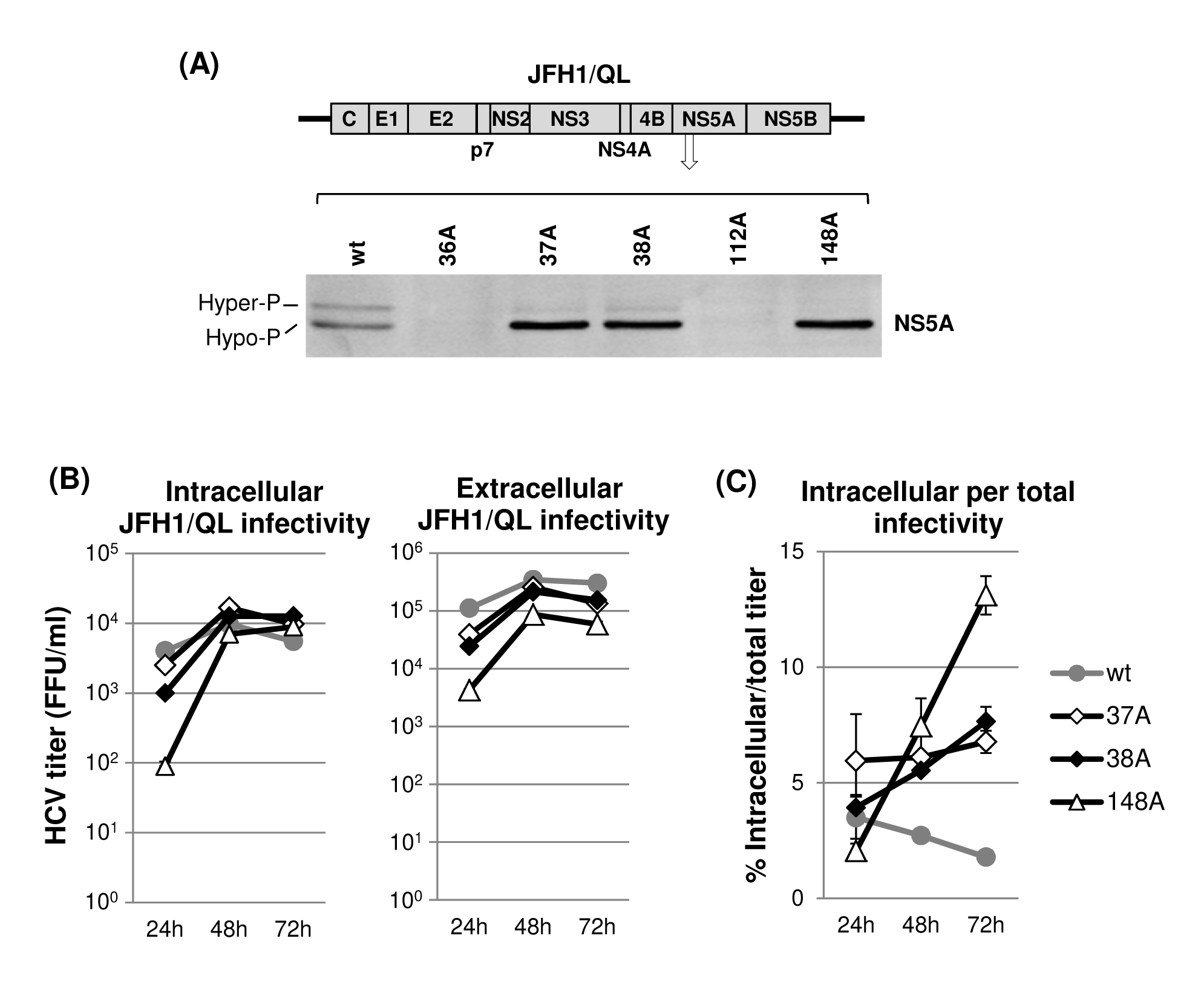

Supplement: S5 Fig — JFH1/QL was generated by introducing cell culture adaptive mutation in NS3 residue Q221L [44]. (A) NS5A western blot analysis of JFH1/QL wt and NS5A dimer interface mutants, which indicates the defective replication of 36A and 112A mutants and also the hyperphosphorylation impairment in 37A, 38A and 148A mutants. (B) Left and right panels show the virus titration results by using cell lysates (intracellular) and cell culture supernatants (extracellular), respectively, at different time points post-electroporation of JFH1/QL RNA with or without indicated mutations. (C) Percentage of intracellular virus titers per total (intracellular plus extracellular) virus titer. (TIF) [file ppat.1007177.s005.tif]

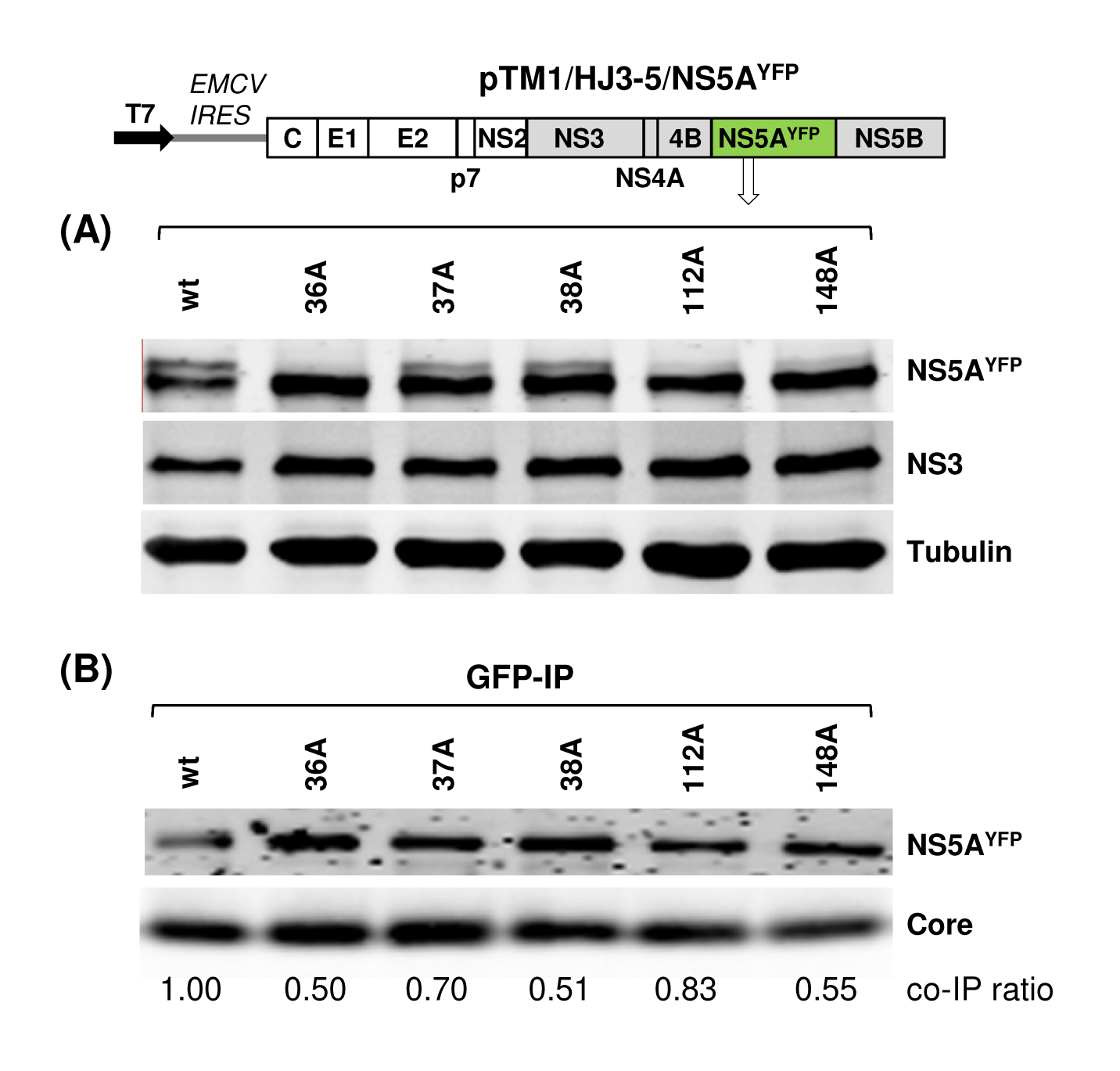

Supplement: S6 Fig — (A) Western blot analysis of NS5AYFP following expression of full length HCV polyprotein derived from HJ3-5/NS5AYFP by using T7 based expression system composed of pTM1 vector ([79], kindly provided by Dr. Bernard Moss at the National Institute of Health, USA) and T7-Lunet cells ([80], kindly provided by Dr. Bartenschlager at the University of Heidelberg, Germany). (B) GFP immunoprecipitation (IP) followed by western blot analysis to detect NS5A and core interaction. Shown at the bottom is the relative NS5A and core co-immunoprecipitation (co-IP) efficiency. (TIF) [file ppat.1007177.s006.tif]

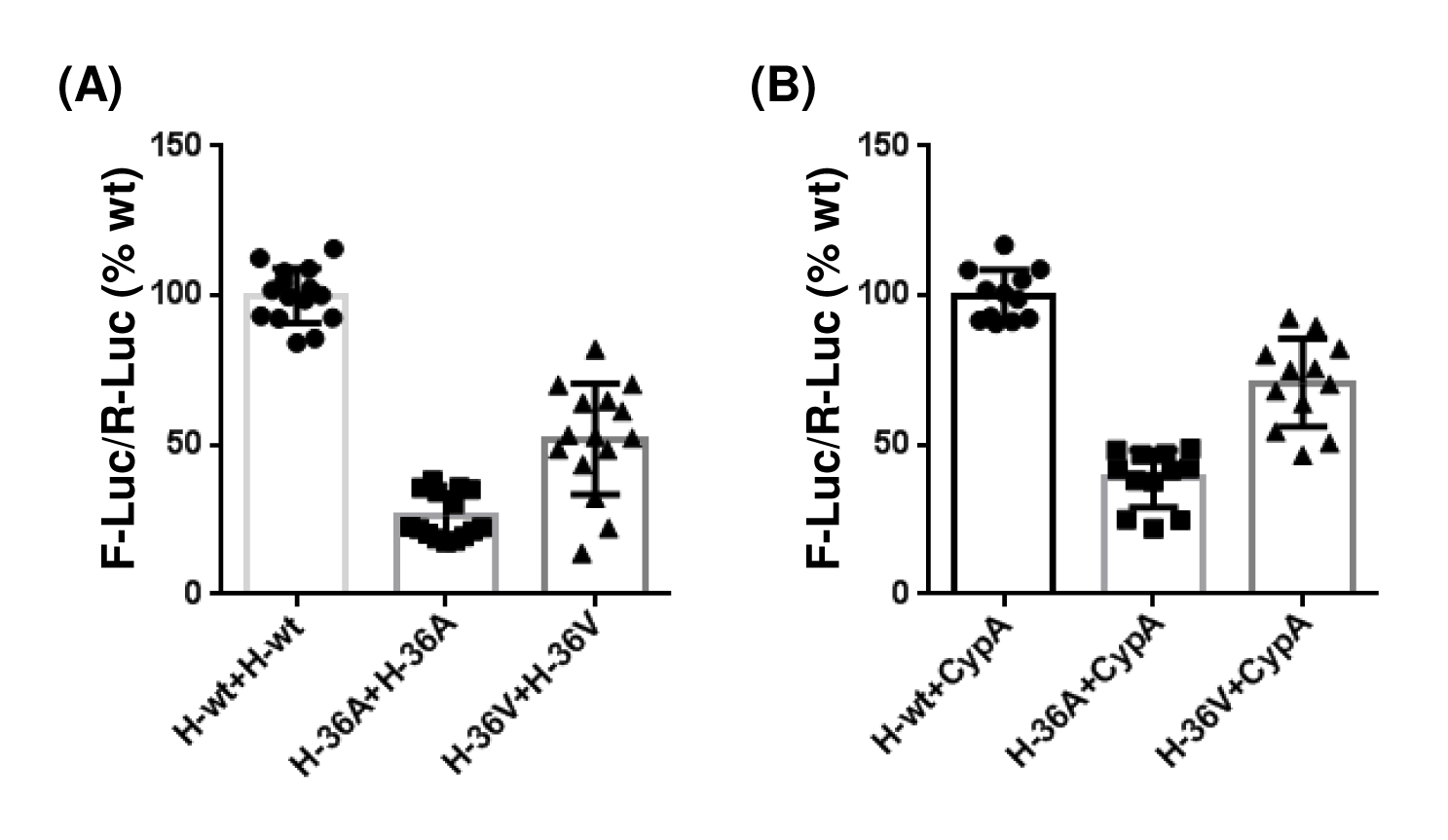

Supplement: S7 Fig — The effects of NS5A/36V mutations on H77 NS5A self-interaction (A) and NS5A-CypA interaction (B) as determined by a checkmate mammalian two-hybrid assay. (TIF) [file ppat.1007177.s007.tif]
